# A Multi-Layered Analytical Pipeline Combining Informatics, UHPLC–MS/MS, Network Pharmacology, and Bioassays for Elucidating the Skin Anti-Aging Activity of *Melampyrum roseum*

**DOI:** 10.3390/ijms262411853

**Published:** 2025-12-08

**Authors:** Min Hyung Cho, JangHo Ha, Haiyan Jin, SoHee An, SungJune Chu

**Affiliations:** 1 Bioinformatics and Molecular Design Research Center (BMDRC), Songdogwahak-ro 85, Yeonsu-gu, Incheon 21983, Republic of Korea; 2 Department of Integrative Biotechnology, Yonsei University, Songdogwahak-ro 85, Yeonsu-gu, Incheon 21983, Republic of Korea

**Keywords:** skin anti-aging, natural products, *Melampyrum roseum*, metabolite profiling, network pharmacology, molecular dynamics

## Abstract

Oxidative stress, UV exposure, inflammation, and extracellular matrix degradation collectively drive skin aging, underscoring the need for safe, multi-target therapeutic options. We developed and applied an integrated analytical pipeline combining UHPLC–MS/MS metabolomics, computational analyses (network pharmacology, molecular docking, and molecular dynamics simulation), and experimental bioassays to efficiently identify and characterize novel natural products with anti-aging potential. This workflow was applied to *Melampyrum roseum* Maxim., a previously unassessed hemiparasitic plant of the Orobanchaceae family, to elucidate its bioactive potential against skin aging. UHPLC–MS/MS profiling annotated 13 secondary metabolites, predominantly flavone aglycones, iridoid glycosides, and phenylpropanoid derivatives. Network pharmacology analysis linked these metabolites to 172 potential skin-aging-associated targets, mainly within inflammatory, ECM, and oxidative-stress pathways. Molecular docking and 100-ns molecular dynamics simulations confirmed stable ligand-target interactions with favorable binding energies, particularly with AKT1, EGFR, PTGS2 and XDH. Validating these predictions, the *M. roseum* extract demonstrated significant antioxidant activity and effectively suppressed key inflammatory mediators (IL-6, TNF-α, COX-2) and MMP-1 levels in UVB-exposed fibroblasts, notably without significant cytotoxicity. Collectively, these findings demonstrate that *M. roseum* harbors multifunctional metabolites that modulate key inflammatory and matrix-regulatory pathways, providing preliminary mechanistic evidence for its potential as a promising candidate for natural anti-aging applications.

## 1. Introduction

Skin aging represents a multifactorial biological process driven by intrinsic and extrinsic factors that progressively impair the structure and function of the skin [1]. Intrinsic aging arises from genetic and metabolic influences that lead to cellular senescence, mitochondrial dysfunction, and gradual loss of collagen homeostasis [2,3,4]. In contrast, extrinsic aging is primarily induced by chronic exposure to ultraviolet (UV) radiation and oxidative stress generated by environmental pollutants, infrared light, and lifestyle-related insults [5]. These stressors activate kinase cascades triggered by epidermal growth factor and cytokine receptor signaling, which in turn promote phosphorylation of pro-inflammatory downstream mediators: the resulting amplification of inflammatory and oxidative pathways accelerates collagen degradation, elastin fragmentation, and extracellular matrix (ECM) remodeling, culminating in visible phenotypes such as reduced elasticity, coarse texture, uneven pigmentation, and wrinkle formation [6,7,8]. Among these factors, UV radiation remains the dominant external contributor: UVA penetrates deeply into the dermis and stimulates reactive oxygen species (ROS) production, while UVB causes direct DNA damage and cytokine-mediated inflammation [6,9]. Sustained activation of these mechanisms causes chronic oxidative injury, immunosuppression, and premature cellular apoptosis, establishing a feed-forward loop of inflammation and ECM breakdown. Clinically, such molecular alterations manifest as dryness, epidermal thickening, pigmentary disorders, and actinic wrinkles, and in severe cases, promote carcinogenesis [10,11,12]. Beyond cosmetic concerns, the cumulative biological and psychological burden of skin aging underscores the need for preventive and therapeutic strategies targeting oxidative and inflammatory signaling at the molecular level.

Despite advances in dermatological and cosmetic science, current interventions provide only partial protection against the multifactorial processes of skin aging. Topical sunscreens and emollients can mitigate ultraviolet exposure and transepidermal water loss but fail to reverse molecular and structural damage once it occurs [13]. Pharmacological agents such as retinoic acid and 5-fluorouracil promote epidermal turnover and collagen synthesis; however, their efficacy is dose-dependent and frequently accompanied by erythema, irritation, and photosensitivity, limiting long-term compliance [14]. Synthetic antioxidants such as vitamins C and E exhibit limited stability and skin penetration, whereas procedural interventions like chemical peels and lasers provide only transient cosmetic improvements [15,16]. Consequently, there is increasing demand for safe, multifunctional agents that modulate the core molecular drivers of oxidative and inflammatory skin aging.

Natural products (NPs) represent one of the richest sources of pharmacologically active compounds and continue to inspire modern drug discovery [17,18]. Their evolutionary “optimization” within biological systems has endowed them with high scaffold diversity, stereochemical complexity, and favorable physicochemical properties such as increased sp^3^ character, rigidity, and hydrophilicity compared with synthetic molecules, thereby enabling selective modulation of protein–protein interactions and signaling cascades that are difficult to target with conventional small molecules [19]. Moreover, NPs occupy a broader and more biologically relevant chemical space, covering structural motifs that have co-evolved with living organisms to maintain homeostasis under oxidative and inflammatory stress [17,20,21]. Such intrinsic bioactivity gives NPs unique potential as senolytic agents capable of attenuating cellular senescence, modulating mitochondrial redox balance, and re-activating endogenous repair pathways [22,23]. Compounds such as resveratrol, quercetin, fisetin, and sulforaphane have demonstrated selective clearance of senescent cells or activation of cytoprotective KEAP1–NRF2 signaling, thereby restoring redox and proteostasis equilibrium [22,23,24,25]. Compared with synthetic analogues, these multi-target natural metabolites exhibit lower cytotoxicity, greater biocompatibility, and synergistic effects through network-level regulation of redox, inflammatory, and extracellular-matrix pathways [26,27]. Consequently, natural products provide a mechanistically versatile and evolutionarily validated reservoir for the discovery of safe and multifunctional anti-aging agents.

Traditional natural-product research, however, relies on labor-intensive extraction, bioactivity-guided fractionation, and structural elucidation, which are inherently time-consuming and often lead to repeated rediscovery of known ingredients [28]. Such workflows provide valuable mechanistic insight but remain poorly suited for the rapid identification of novel bioactives within chemically complex matrices. Recent advances in analytical instrumentation, database availability, and computational modeling have begun to transform this landscape [29]. Expanding public resources—such as GNPS, METLIN, and molecular-networking repositories—now enable large-scale comparison of metabolomic fingerprints, while in silico methodologies including network pharmacology, molecular docking, and molecular-dynamics simulations can predict biological targets and estimate binding stability prior to experimental validation [30,31]. Nevertheless, integrating heterogeneous data types across chemical, biological, and pharmacological domains remains challenging. The quality, completeness, and interoperability of available datasets vary widely, complicating the construction of reproducible predictive models [32]. To overcome these limitations, an integrative analytical pipeline combining multidimensional information has emerged as a powerful strategy. By linking metabolomic annotation with systems-level network analysis and computational validation, such a pipeline allows consistent evaluation of diverse natural materials, streamlines sequential screening, and reduces both time and resource requirements. This data-driven framework enables prioritization of genuine natural products with high mechanistic relevance rather than reliance on empirical screening alone [33,34]. Ultimately, coupling informatics-based prediction with experimental corroboration establishes a scalable foundation for rational natural-product discovery—transforming traditional pharmacognosy into an efficient, knowledge-driven discipline.

**Figure 1:**
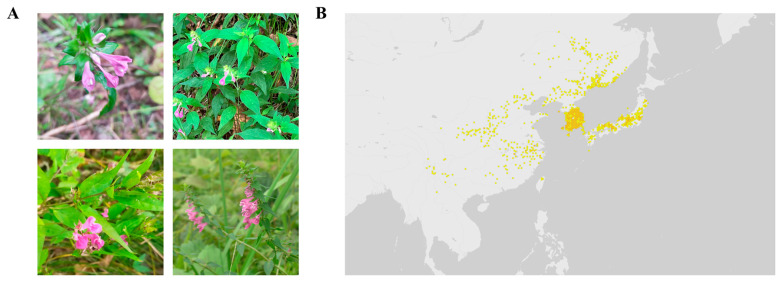
Botanical characterization of *M. roseum*. (**A**) Assorted images of *M. roseum* in its natural habitat. Retrived from iNaturalist.org (Photo IDs: 101576736, 156567394, 232793953, 540104974, https://www.inaturalist.org, accessed on 5 December 2025). (**B**) Native distribution of *M. roseum*. Retrieved from GBIF Georeferenced records. Yellow dots: georeferenced point records representing individual occurrences.

Within this framework, we investigated plant species native to the Korean Peninsula and neighboring East Asian regions to identify phytochemicals with potential anti-aging effects. Among them, *Melampyrum roseum* (Figure 1) emerged as a particularly promising candidate. *M. roseum* is a hemiparasitic herb belonging to the family Orobanchaceae, distributed across East Asia, including China, the Korean Peninsula, Japan, Taiwan, and the Russian Far East [35]. The plant typically inhabits temperate grasslands and forest margins, forming partial parasitic associations with nearby host roots. Despite its ecological adaptability and taxonomic distinctiveness, the species has remained almost entirely unexplored in pharmacological or phytochemical contexts. Notably, phylogenetically related taxa within Melampyrum and the broader Orobanchaceae—such as Euphrasia, Pedicularis, and Rehmannia—are well recognized for producing diverse iridoids, phenylethanoid glycosides, lignans, and flavonoids with potent antioxidant, neuroprotective, and anti-inflammatory activities [36,37,38]. These chemotaxonomic parallels suggest that *M. roseum* may share biosynthetic pathways yielding structurally diverse metabolites capable of modulating redox and inflammatory signaling relevant to skin aging.

**Figure 2:**
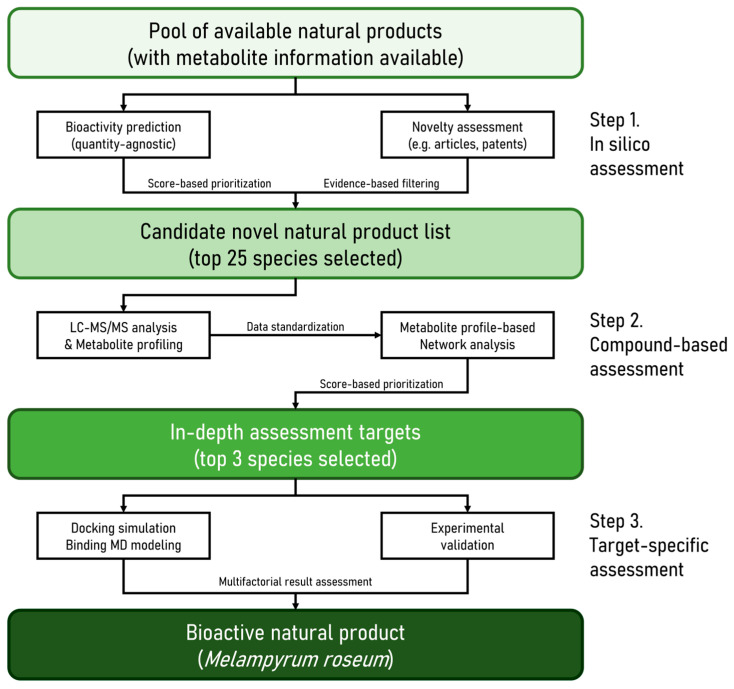
Workflow diagram for identification of *M. roseum* as a novel active natural product.

Building upon this rationale, the present study aimed to elucidate the chemical composition and molecular basis underlying the potential skin anti-aging activity of *M. roseum* (Figure 2). To achieve this, we implemented a multi-layered analytical pipeline that integrates experimental and computational methodologies for comprehensive natural-product evaluation. The workflow comprised (i) identification and sourcing of *M. roseum*, (ii) untargeted UHPLC–MS/MS metabolomic profiling and compound annotation, (iii) network pharmacology to identify skin-aging-related targets, (iv) molecular docking and molecular-dynamics simulations to predict ligand–target interactions and binding stability, and (v) in vitro bioassays using human dermal fibroblasts to validate antioxidant and anti-inflammatory effects. This integrative approach enabled systematic prioritization of bioactive metabolites and mechanistic correlation with key signaling pathways implicated in inflammation and extracellular matrix remodeling. Our findings revealed that *M. roseum* contains diverse secondary metabolites—principally flavonoids, iridoids, and phenylethanoid glycosides—that exert antioxidative and anti-inflammatory activities, effectively suppressing IL-6, TNF-α, COX-2, and MMP-1 expression in UVB-exposed fibroblasts. Collectively, this study establishes *M. roseum* as a previously unrecognized source of multifunctional compounds and demonstrates the utility of our integrated informatics-to-bioassay pipeline for data-driven natural-product discovery.

## 2. Results

### 2.1. Metabolite Profiling of M. roseum Extract Using UHPLC-MS/MS

To identify the main chemical constituents of the *M. roseum* extract, a metabolite profiling analysis was conducted using a UHPLC-Orbitrap ID-X Tribrid mass spectrometer. The extract (1 mg/mL) was analyzed in both positive (+ESI) and negative (−ESI) ion modes to ensure comprehensive detection of diverse chemical classes.

**Figure 3:**
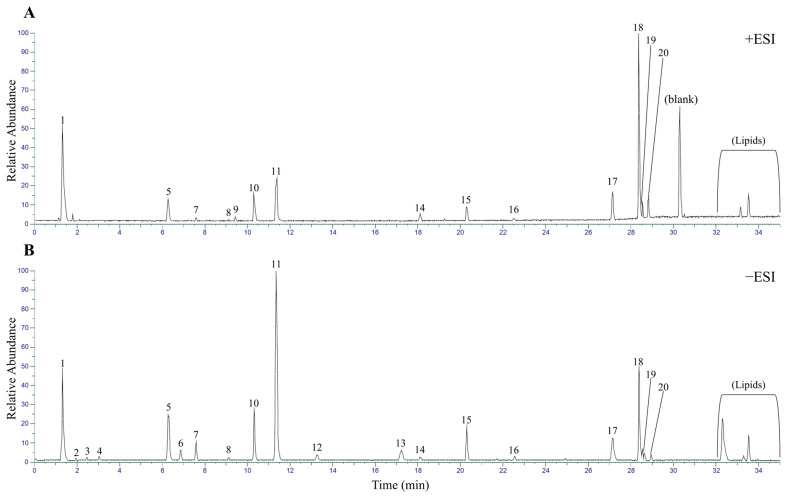
Base peak chromatograms of *M. roseum* extract using UHPLC-Orbitrap-MS/MS. (**A**) Positive ESI mode. (**B**) Negative ESI mode.

The base peak chromatograms (BPCs) obtained from the analysis are presented in Figure 3. Both positive (Figure 3A) and negative (Figure 3B) ionization modes displayed numerous distinct peaks across the entire 35-min chromatographic run, indicating that the *M. roseum* extract is a complex mixture containing a wide variety of secondary metabolites. By comparing with blank sample, we were able to identify 20 significant peaks, which were subjected to further analysis using data-dependent acquisition (DDA) for MS/MS fragmentation. The high-resolution mass data (Full MS scan at 120,000 resolution) and the resulting MS/MS fragmentation patterns were used to tentatively identify the chemical structures of the metabolites present in the *M. roseum* extract. We also observed that the metabolite profiles were largely consistent among independently collected *M. roseum* samples, supporting the robustness and reproducibility of the extraction and analysis (Supplementary Figure S1). The identified compounds are summarized in Supplementary Table S1.

### 2.2. Identification and Classification of Putative Bioactive Metabolites

The untargeted UHPLC–MS/MS profiling of *M. roseum* extract revealed a diverse spectrum of metabolites encompassing both primary and secondary metabolic classes. The raw dataset included simple sugars, organic acids, fatty acids, and phospholipids, which were systematically excluded to focus on structurally specific secondary metabolites with higher biological relevance. After refinement, thirteen major metabolites were confidently attributed to *M. roseum* with an acceptable confidence level (Level 2a: probable structure based on MS, MS/MS, and library/bibliography search) (Table 1, Supplementary Figure S2).

These compounds were categorized into three principal scaffold classes: flavonoid, iridoid glycoside and phenylpropanoid glycoside. Among them, mussaenoside, apigenin, loganin, loganic acid, (iso)verbascoside and luteolin constituted the predominant fraction, consistent with their strong chromatographic signals and characteristic fragmentation patterns. Other flavonoid and iridoid derivatives were detected with lower relative abundance, suggesting their secondary contribution to the extract’s overall bioactivity. Because of isomeric overlap and limited chromatographic resolution under the present analytical settings, the specific positional isomers of verbascoside and related compounds could not be unequivocally distinguished.

### 2.3. Identification of Mechanistic Targets Involved in Skin Aging

**Figure 4:**
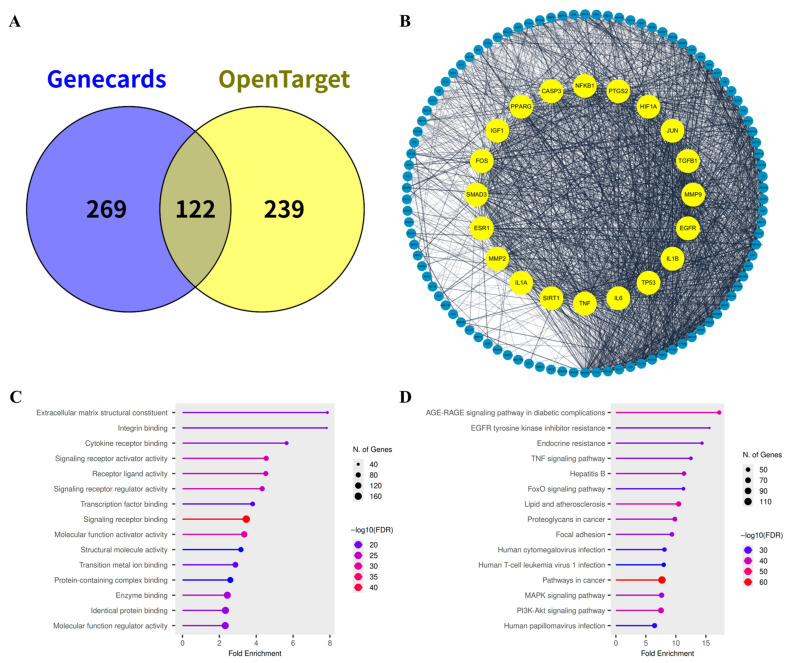
Network profile of skin aging-related genes. (**A**) Venn diagram showing the intersection between disease-associated targets obtained from the GeneCards (blue) and Open Targets (yellow) databases. (**B**) Protein–protein interaction (PPI) network of 122 intersecting genes, highlighting the top 20 hub genes in yellow. (**C**) Gene Ontology (GO) molecular function (MF) enrichment analysis of the 122 intersecting genes. (**D**) KEGG pathway enrichment analysis of the 122 intersecting genes.

To delineate molecular targets mechanistically implicated in skin aging, we queried the GeneCards and Open Targets databases using the query “skin aging.” A total of 630 non-redundant targets were retrieved (Supplementary Table S2). Cross-validation of the two datasets identified 122 overlapping genes (Figure 4A), representing high-confidence targets with consistent disease associations.

A protein–protein interaction (PPI) network comprising 122 overlapping genes was subsequently constructed using the STRING platform (Figure 4B, Supplementary Table S3). Topological analysis of the network revealed that the skin-aging interactome is organized around several functional hubs. Among the top 20 nodes ranked by degree centrality, prominent clusters included pro-inflammatory cytokines (*TNF*, *IL6*, *IL1A*, and *IL1B*), growth factors and receptors (*EGFR*, *TGFB1*, *IGF1*, *SMAD3*), extracellular matrix–degrading enzymes (*MMP2*, *MMP9*), transcriptional regulators (*JUN*, *FOS*, *NFKB1*), and cell death–associated mediators (*TP53*, *CASP3*). These hub proteins collectively represent signaling convergence points governing inflammation, cellular senescence, and ECM remodeling during cutaneous aging.

Functional enrichment was performed on the 122 shared targets using the ShinyGO v0.85 platform. A total of 1795 enriched terms (1000 for GO biological processes (BP), 176 for GO cellular components (CC), 421 for GO molecular functions (MF), 198 for KEGG) were obtained (Supplementary Table S4), from which the top 15 categories were selected for visualization (Figure 4C,D, Supplementary Figure S3). GO enrichment analysis highlighted pathways related to extracellular matrix structural constituents, signaling receptor activity, transcription factor binding, and metal ion binding. Correspondingly, KEGG pathway analysis revealed significant enrichment in EGFR signaling, TNF signaling, MAPK signaling, and PI3K–Akt signaling—canonical cascades known to coordinate oxidative stress responses, inflammatory activation, and collagen turnover in senescent skin.

### 2.4. Identification of Potential Functional Targets of M. roseum Metabolites

**Figure 5:**
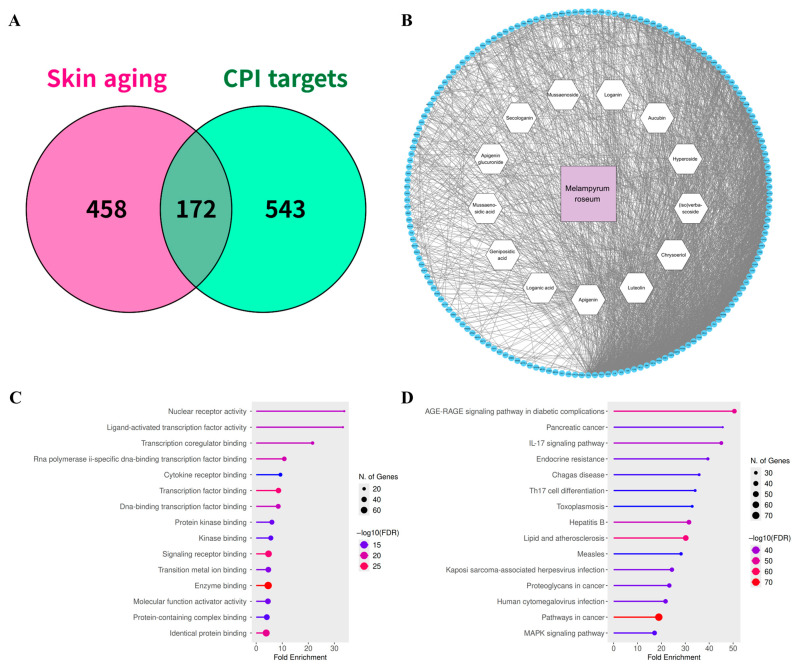
Network pharmacology to analyze *M. roseum* extract for skin aging. (**A**) Venn diagram showing the intersection between non-redundant disease-associated targets collected from databases (magenta) and compound-associated targets (green). (**B**) Compound-protein interaction (CPI) and protein-protein interaction (PPI) network of 13 *M. roseum* secondary metabolites and 172 intersecting genes. (**C**) Gene Ontology (GO) molecular function (MF) enrichment analysis of the 172 intersecting genes. (**D**) KEGG pathway enrichment analysis of the 172 intersecting genes.

Integrating experimentally reported and computationally predicted compound–protein interaction data, we identified 1346 distinct compound-target pairs which involve 715 non-redundant targets and 13 secondary metabolites detected in *M. roseum* extract. These data were compiled from PubChem, CTD, DGIdb, BindingDB, SwissTargetPrediction, and STITCH, ensuring broad coverage of validated and inferred molecular interactions (Supplementary Tables S5–S8). Comparison with the skin-aging–related gene set obtained from GeneCards and Open Targets revealed 172 overlapping targets (Figure 5A, Supplementary Table S9), representing putative functional mediators through which *M. roseum* metabolites may exert anti-aging activity.

A compound–protein interaction network was similarly constructed using Cytoscape v3.10.4, where node degree and betweenness centrality were used to assess topological importance (Figure 5B). The resulting network displayed a characteristic ‘multi-compound–multi-target’ configuration, suggesting synergistic or complementary mechanisms typical of natural-product mixtures. Among the detected constituents, flavone aglycones (apigenin, luteolin, and chrysoeriol) exhibited the highest target connectivity, followed by phenylethanoid glycosides ((iso)verbascoside) and iridoid glycosides (loganin, loganic acid, and secologanin), which was generally consistent with the number of scientific reports associated with each metabolite scaffold class (Supplementary Table S10).

### 2.5. Network Pharmacology & Pathway Enrichment Analysis

Analysis of the PPI network, constructed from the 172 intersecting targets using the STRING database (confidence score ≥ 0.7), revealed key nodes associated with inflammatory signaling, oxidative stress regulation, and extracellular matrix (ECM) remodeling. The top ten hub targets, ranked by degree centrality, were *TNF*, *IL6*, *TP53*, *AKT1*, *IL1B*, *JUN*, *CASP3*, *STAT3*, *HIF1A* and *EGFR*. All aforementioned targets are primarily involved in cellular signaling, structural maintenance and cell death, notably within the PI3K–Akt, STAT3, and ECM-degradation pathways—core regulatory axes implicated in skin aging.

To elucidate the biological functions and signaling relevance of the intersecting targets between *M. roseum* metabolites and skin-aging–related genes, GO and Kyoto Encyclopedia of Genes and Genomes (KEGG) enrichment analyses were performed using the ShinyGO v0.85 platform. Enrichment results were filtered using a false discovery rate (FDR) < 0.05, and the top-ranked terms and pathways were visualized for clarity (Figure 5C,D, Supplementary Figure S4).

The GO enrichment analysis revealed over 1000 biological processes (BP), 139 cellular components (CC), and 283 molecular functions (MF) significantly associated with the target set (Supplementary Table S11). Among the BP categories, processes related to response to chemical and oxidative stress, prostaglandin and cytokine-mediated signaling, regulation of cell motility, and cell survival and proliferation were highly enriched, reflecting the multifactorial mechanisms underlying skin aging. The CC terms were primarily enriched in the extracellular matrix, plasma membrane, and nuclear compartments, suggesting that *M. roseum* metabolites influence both extracellular structural remodeling and intracellular stress responses. The MF categories were dominated by membrane or nuclear receptor binding, enzyme binding, and transcription factor interaction, functions essential for maintaining homeostatic control over redox balance and signal transduction.

The KEGG pathway analysis identified 207 significantly enriched pathways, several of which were directly linked to inflammation, oxidative stress, and matrix degradation—key pathophysiological features of skin aging (Supplementary Table S11). Notably, pathways such as AGE–RAGE signaling, IL-17 signaling, EGFR signaling, and Th17 cell differentiation were prominently represented. In addition, multiple enriched pathways were related to cell survival and tumor-related signaling (e.g., PI3K–Akt, MAPK, and apoptosis regulation), suggesting that *M. roseum* metabolites may modulate universal cytoprotective mechanisms. Interestingly, a subset of enriched pathways corresponded to infectious or immune-mediated disorders, including trypanosomiasis, leishmaniasis, legionellosis, malaria, and toxoplasmosis, indicating potential overlap between immune regulation and anti-inflammatory processes engaged by these metabolites.

Collectively, this multi-layered network analysis and pathway enrichment analysis underscores the poly-pharmacological potential of *M. roseum* metabolites, delineating a biologically coherent set of targets that may cooperatively modulate oxidative balance, inflammatory responses, and dermal matrix integrity.

### 2.6. Molecular Docking and Dynamics Validation

#### 2.6.1. Molecular Docking

**Figure 6:**
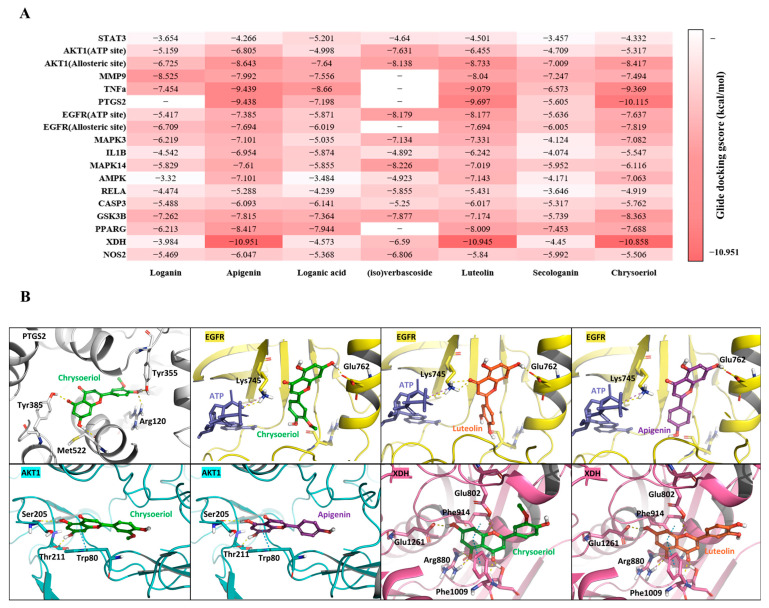
Molecular docking analysis of selected compounds against key targets. (**A**) Heatmap of Glide docking gscores (kcal/mol) for seven natural compounds against 18 protein targets. (**B**) Representative docking structures of Chrysoeriol, Luteolin, and Apigenin against four targets with favorable interaction profiles. PTGS2, EGFR, AKT1, and XDH are depicted as white, yellow, cyan, and pink ribbons, respectively. Chrysoeriol, Luteolin, and Apigenin are shown as green, orange, and purple sticks, respectively. Key interacting residues forming hydrogen bonds or hydrophobic contacts with the ligands are labeled.

To further validate the network pharmacology results, molecular docking analyses were conducted between the seven *M. roseum*-derived natural compounds and a total of sixteen target proteins identified from the integrated network (Figure 6A). Given their distinct regulatory pockets, both the ATP-binding and allosteric sites of EGFR and AKT1 were evaluated. Among the tested compounds, Chrysoeriol, Luteolin, and Apigenin exhibited comparatively favorable Glide docking scores particularly against PTGS2, the EGFR allosteric site, the AKT1 allosteric site, and XDH. Visual inspection of the docking poses confirmed that these ligands established stable binding modes by maintaining key interactions with essential residues in each protein binding pocket (Figure 6B).

#### 2.6.2. MD Simulation

**Figure 7:**
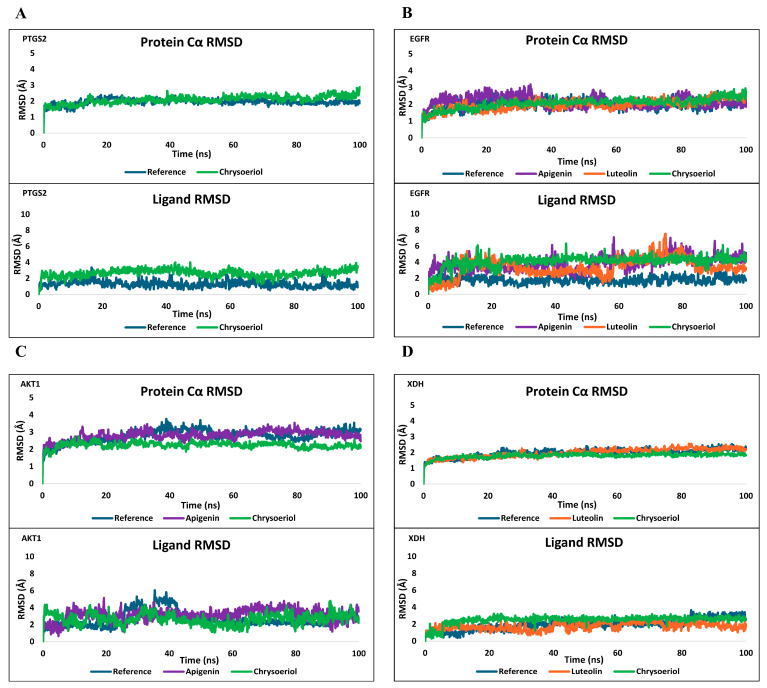
Molecular dynamics simulation results of three compounds bound to target proteins (replicate 1). Protein Cα and ligand RMSD trajectories over the 100 ns simulation are presented for the protein–ligand complexes. The reference ligand corresponds to the co-crystallized ligand from each target protein structure. Chrysoeriol, Luteolin, and Apigenin are represented in green, orange, and purple traces, respectively. (**A**) PTGS2. (**B**) EGFR. (**C**) AKT1. (**D**) XDH.

Subsequently, the eight protein–ligand complexes identified from the docking analysis were subjected to 100 ns all-atom MD simulations for further validation. The MD simulation analysis results are summarized in Figure 7 and Figure S1. Across all targets, protein Cα RMSD remained stable when compared with reference ligand complexes, indicating preserved global structural integrity. Ligand RMSD profiles revealed that compound binding within AKT1 and XDH was highly consistent throughout the simulation, comparable to the stability observed with the co-crystallized ligands. Although PTGS2 and EGFR exhibited slightly higher fluctuations, the overall RMSD values remained within approximately 5 Å, suggesting that deviations were not significant enough to disrupt ligand retention in their respective pockets.

Across the three simulation replicas (Figure 7, Supplementary Figures S5 and S6), the protein Cα RMSD profiles exhibited consistent patterns, indicating that all protein–ligand complexes maintained stable conformations throughout the trajectories. Ligand RMSD values were also generally similar across runs, though the third AKT1 replica showed slightly increased fluctuations. Despite this, the ligand remained confined within the binding pocket. The XDH complex displayed highly reproducible RMSD trends across all replicas, suggesting the strongest and most consistent ligand-binding stability among the four analyzed targets.

Protein–ligand contact analyses further supported these stability outcomes. As shown in Supplementary Figure S7, essential interactions such as hydrogen bonds and hydrophobic contacts persisted for more than 30% of the simulation time in all complexes, indicating sustained intermolecular recognition during the dynamics process. Collectively, the docking and MD simulation results support that Chrysoeriol, Luteolin, and Apigenin possess strong structural compatibility and durable binding interactions with PTGS2, EGFR allosteric site, AKT1 allosteric site, and XDH, highlighting their potential as multi-target agents.

### 2.7. Experimental Validation

#### 2.7.1. Cell Viability Assessment

**Figure 8:**
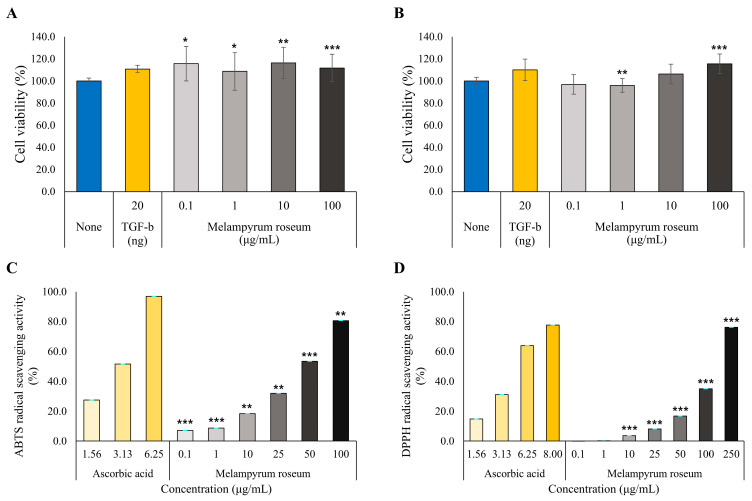
Effects of *M. roseum* extract on cell viability and free radical scavenging. Data are expressed as the mean ± S.D of three replicates. Note that some error bars are smaller than the symbols/lines. *** *p* < 0.001, ** *p* < 0.01 and * *p* < 0.05 as compared to none. (**A**) Effects of *M. roseum* extract on RAW264.7 cell viability. (**B**) Effects of *M. roseum* extract on human dermal fibroblasts (HDFs) cell viability. (**C**) ABTS radical scavenging activity of *M. roseum* extract. (**D**) DPPH radical scavenging activity of *M. roseum* extract.

Prior to evaluating the anti-inflammatory (in RAW264.7 cells) and anti-wrinkle (in HDFs) effects of the *M. roseum* extract, an MTT assay was performed to determine its effect on cell viability (Figure 8A,B). No cytotoxicity was observed at any of the tested concentrations: actually, in RAW264.7 cell model, overall cell viability has increased slightly but significantly. Likewise, in HDF cell model, increase of cell viability was observed in 10 µg/mL and 100 µg/mL condition. Based on these results, it was confirmed that the *M. roseum* extract is non-toxic to both cell lines at concentrations up to 100 µg/mL. Therefore, all subsequent efficacy experiments were conducted within this concentration range.

#### 2.7.2. Antioxidant Activity Assessment

To evaluate the antioxidant efficacy of the *M. roseum* extract, the representative radical scavenging assays, ABTS and DPPH, were performed. Ascorbic acid was used as a positive control.

The *M. roseum* extract effectively scavenged ABTS radicals in a concentration-dependent manner (Figure 8C). Starting from a 7.2% scavenging activity at a low concentration of 0.1 µg/mL, the activity significantly increased as the concentration rose: 8.7% (1 µg/mL), 18.5% (10 µg/mL), 32.0% (25 µg/mL), 53.4% (50 µg/mL), and 80.6% (100 µg/mL) (*p* < 0.01 or *p* < 0.001 at all concentrations). The IC50 value of the *M. roseum* extract was calculated to be 53.49 µg/mL. Although this value is higher than the IC50 value of the positive control, Ascorbic acid (3.03 µg/mL), it indicates that the *M. roseum* extract possesses strong ABTS radical scavenging activity at just 53.49 µg/mL, despite being a complex mixture.

In the DPPH radical scavenging activity assay, the *M. roseum* extract also demonstrated a concentration-dependent antioxidant effect (Figure 8D). The extract significantly scavenged DPPH radicals as the concentration increased: 3.7% (10 µg/mL), 8.1% (25 µg/mL), 16.9% (50 µg/mL), 35.1% (100 µg/mL), and 76.2% (250 µg/mL) (*p* < 0.001 at all concentrations from 10 µg/mL upwards). The IC50 value of the *M. roseum* extract was calculated to be 160.02 µg/mL. In comparison, the positive control, Ascorbic acid, showed stronger activity with an IC50 value of 4.51 µg/mL.

Collectively, these results confirm that the *M. roseum* extract possesses concentration-dependent in vitro antioxidant activity in both assays. The IC50 values of the *M. roseum* extract (ABTS: 53.49 µg/mL; DPPH: 160.02 µg/mL) were numerically higher than those of the positive control, Ascorbic acid, a pure single compound (ABTS: 3.03 µg/mL; DPPH: 4.51 µg/mL). However, this is a comparison between a crude extract, which includes a large amount of non-antioxidant components (e.g., sugars, lipids), and a pure compound. The fact that the crude extract exhibited these IC50 values in its unrefined state suggests that the active constituents within *M. roseum* possess strong antioxidant potential.

#### 2.7.3. Anti-Inflammatory Activity Assessment

**Figure 9:**
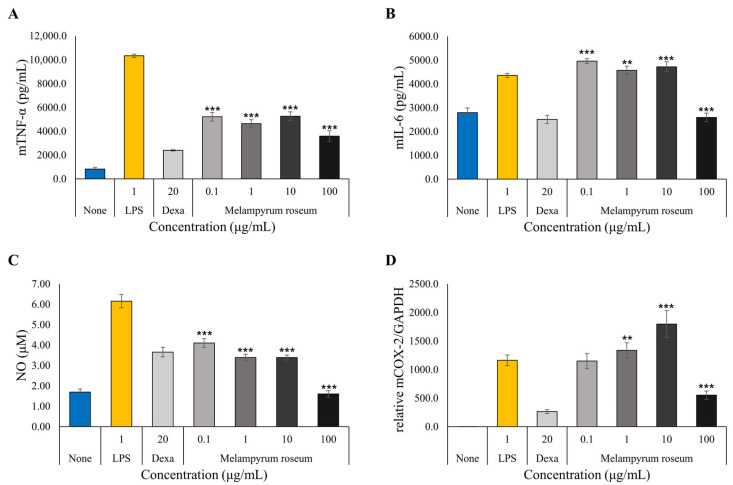
Anti-inflammatory effects of *M. roseum* extract on lipopolysaccharide (LPS)-stimulated RAW264.7 macrophages, demonstrating the inhibition of pro-inflammatory mediators. Data are expressed as the mean ± S.D of three replicates. *** *p* < 0.001, ** *p* < 0.01 as compared to the LPS (1 ug/mL) group. (**A**) TNF-α. (**B**) IL-6. (**C**) NO. (**D**) PTGS2 (COX-2).

The anti-inflammatory activity of *M. roseum* extract was evaluated in LPS-stimulated RAW 264.7 macrophages by measuring its effects on pro-inflammatory cytokines and inflammatory mediators (Figure 9). Dexamethasone (Dexa) was used as the positive control. LPS treatment (1 µg/mL) markedly increased the secretion of TNF-α and IL-6, core pro-inflammatory cytokines, compared with untreated cells, confirming successful induction of inflammation. The extract significantly inhibited TNF-α production at all tested concentrations (0.1–100 µg/mL, *p* < 0.001) in a clear dose-dependent manner (Figure 9A). At 100 µg/mL, TNF-α secretion was reduced to levels comparable to those observed with Dexa, indicating potent inhibitory efficacy. In contrast, IL-6 suppression was evident only at 100 µg/mL (*p* < 0.001), whereas lower concentrations produced a mild stimulatory effect (Figure 9B).

The extract also strongly attenuated nitric oxide (NO) generation in LPS-stimulated cells, exhibiting significant inhibition across all tested concentrations (*p* < 0.001). At concentrations ≥ 1 µg/mL, NO levels were reduced more effectively than by Dexa, approaching near-baseline values at 100 µg/mL. In parallel, qRT-PCR analysis showed that cyclooxygenase-2 (COX-2 or PTGS2) mRNA expression was suppressed by approximately 50% at 100 µg/mL relative to the LPS group (*p* < 0.001), but increased by about 55% at 10 µg/mL. Collectively, *M. roseum* extract exhibited robust, dose-dependent suppression of TNF-α and NO, together with partial modulation of IL-6 and COX-2, demonstrating pronounced anti-inflammatory potential through the coordinated inhibition of multiple mediators and signaling pathways.

#### 2.7.4. Anti-Photoaging Effect Assessment: Inhibition of MMP-1 Production

**Figure 10:**
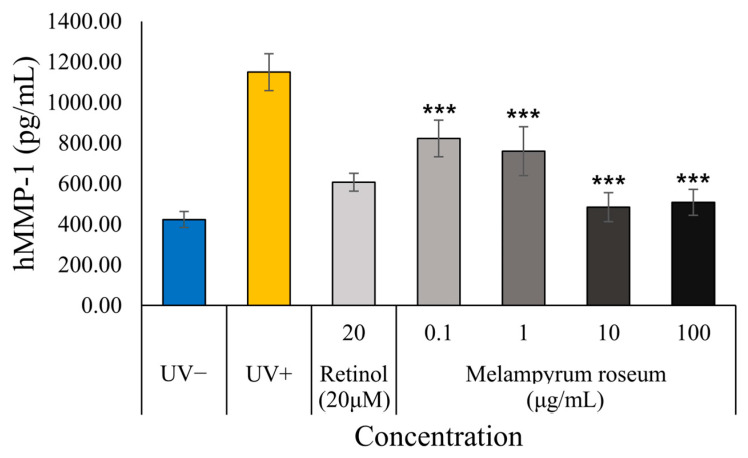
Inhibitory effect of *M. roseum* extract on UVB-induced MMP-1 production in human dermal fibroblasts (HDFs). Data are expressed as the mean ± S.D of three replicates. *** *p* < 0.001 compared to the UV+ group.

The anti-photoaging potential of *M. roseum* extract was evaluated in UVB-irradiated human dermal fibroblasts (HDFs) by measuring the production of matrix metalloproteinase-1 (MMP-1), a key collagen-degrading enzyme (Figure 10). UVB exposure (20 mJ cm^−2^) markedly increased MMP-1 secretion compared with the non-irradiated control, confirming successful induction of photoaging. Treatment with retinol (20 µM), used as a positive control, significantly reduced MMP-1 expression.

The *M. roseum* extract inhibited UVB-induced MMP-1 production in a dose-dependent manner across all tested concentrations (0.1–100 µg mL^−1^, *p* < 0.001 vs. UVB control). Notably, treatment with 10 µg mL^−1^ and 100 µg mL^−1^ extract reduced MMP-1 to levels comparable to or lower than those observed in the retinol group, approaching baseline values of the non-irradiated control. These findings demonstrate that *M. roseum* extract effectively suppresses UVB-induced MMP-1 overexpression, thereby mitigating collagen degradation and suggesting a strong anti-photoaging and anti-wrinkle potential through inhibition of ECM breakdown.

## 3. Discussion

This study highlights the efficacy of an integrated analytical framework that combines untargeted metabolomics, network pharmacology, molecular modeling, and experimental validation to delineate the bioactive landscape of *Melampyrum roseum*. Through this multi-layered approach, we established a direct link between the plant’s chemical diversity and its biological relevance, enabling efficient identification of anti-skin-aging constituents from a previously uncharacterized botanical source. Comprehensive UHPLC–MS/MS profiling and computational prioritization identified flavone aglycones—apigenin, luteolin, and chrysoeriol—to be the principal metabolites associated with the observed bioactivities. These compounds consistently emerged as top-ranked candidates across all analytical layers, exhibiting potent anti-inflammatory and extracellular-matrix–protective effects in silico and in vitro. Molecular docking and 100-ns molecular-dynamics simulations revealed stable binding of these flavonoids to AKT1 and EGFR, forming hydrogen-bonding and π–π stacking interactions within the allosteric cleft near the AKT1 PH domain and overlapping residues of the EGFR allosteric pocket, consistent with a potential role as AKT1/EGFR allosteric modulator. This dual modulation is highly pertinent to the skin-aging context, wherein AKT1-driven oxidative imbalance and EGFR-mediated hyperproliferation synergistically disrupt collagen integrity and dermal homeostasis. Additionally, enzyme-target analyses suggested that these flavonoid aglycones could exert supplementary inhibitory activity toward COX-2 and XDH, both key mediators of inflammatory and oxidative stress responses.

Pathway enrichment linked the predicted targets to PI3K–Akt, MAPK, and NF-κB signaling cascades, central regulators of cell survival, cytokine production, and collagen turnover (Figure 5). Inhibition of these pathways may attenuate UV-induced inflammatory responses, reduce MMP-1 activation, and preserve dermal matrix integrity [39,40]. Inhibition of MAPK14 may further prevent NF-κB nuclear translocation via blocking JNK phosphorylation, thereby reducing cytokine transcription and limiting matrix metalloproteinase activation. Although flavonoids such as apigenin and luteolin are widely studied anti-inflammatory phytochemicals [41,42], our findings provide additional insight into how their specific combination within *M. roseum* may confer synergistic protection in cutaneous systems. Molecular dynamics simulation suggested that these compounds converge on overlapping yet distinct target sets (Figure 7): apigenin displayed strong affinity for AKT1 and EGFR, luteolin interacted preferentially with EGFR and XDH, whereas chrysoeriol bridged both groups through interactions with AKT1, COX-2 and XDH. Importantly, these predicted interactions align with established biochemical evidence; prior studies have validated luteolin as an ATP-competitive inhibitor of EGFR and a suppressor of AKT signaling [43,44], while apigenin and chrysoeriol are documented to inhibit AKT phosphorylation and downstream PI3K/AKT cascades [45,46]. Such distributed target engagement may underlie the extract’s ability to simultaneously modulate oxidative and inflammatory stress, an effect greater than would be expected from any single constituent.

It is noteworthy that the high degree centrality of flavone aglycones in our network analysis partially reflects a ‘knowledge bias.’ As confirmed by our bibliometric analysis (Supplementary Table S10), flavonoids like apigenin and luteolin have been subject to extensive pharmacological research compared to iridoids such as loganin. Since network pharmacology relies on existing interaction databases, these well-studied compounds naturally exhibit higher connectivity. Nevertheless, this alignment with previous literature reinforces the plausibility of their bioactive roles [47,48,49].

Although the free-radical scavenging activity of the *M. roseum* extract did not substantially exceed high-potency benchmarks, this outcome is consistent with the predominance of metabolites with relatively mediocre radical scavenging potential such as apigenin, chrysoeriol and loganin in the extract [50,51] (Supplementary Table S13). These compounds have been documented to exert antioxidant effects via activation of endogenous defense systems—such as Nrf2 pathway up-regulation—in addition to direct radical neutralization [52]. Furthermore, our molecular-dynamics results include stable interactions of chrysoeriol and luteolin with XDH/Xanthine oxidase (XO), suggesting that *M. roseum* metabolites may suppress ROS generation by inhibiting a key source enzyme rather than relying solely on high radical-scavenging kinetics [53,54]. These dual mechanisms—moderate direct scavenging plus enzyme-mediated reduction of oxidative burden—may contribute to greater chemical stability and dermal relevance in a complex biological environment.

A notable biphasic response was observed in the anti-inflammatory assays for IL-6 and COX-2 (Figure 9A,C), which were stimulated at low concentrations but significantly suppressed at 100 µg/mL. This phenomenon is consistent with hormesis, where low-dose phytochemicals can act as mild cellular primers [55,56]. However, the key upstream mediators, TNF-α and NO (Figure 9B,D), were potently and consistently inhibited across all concentrations in a dose-dependent manner. This strongly suggests that the primary anti-inflammatory mechanism is the robust suppression of the TNF-α/NO axis [57], which becomes dominant at higher concentrations to subsequently inhibit the downstream IL-6 and COX-2 pathways.

Compared with previous studies on plant-derived anti-aging or photoprotective agents, the present work differs fundamentally in both methodological design and conceptual scope. Traditionally, natural product research has relied on bioassay-guided fractionation or targeted compound testing, approaches that are effective for isolating individual actives but inherently limited when applied to chemically complex botanical systems [26]. Such reductionist workflows often lead to the repeated identification of known flavonoids or phenolics, while offering little guidance on the selection of promising plant sources at the outset [28]. Recent studies employing network pharmacology and molecular docking have partially addressed the mechanistic gap by linking compounds to putative biological targets in silico; however, these methods typically operate after a specific plant or extract has already been chosen, thus providing limited value in the initial prioritization of candidate species [31,33,34]. In contrast, our workflow establishes a data-driven “from-selection-to-validation” pipeline, beginning with information-based pre-evaluation of multiple botanical candidates, followed by untargeted UHPLC–MS/MS metabolomics, network pharmacology, and molecular-dynamics simulations. This top-down predictive route connects chemical identity to biological function prior to empirical testing, allowing efficient prioritization of plants and metabolites most likely to exhibit anti-aging activity. By integrating quantitative metabolomic signatures with systems-level target prediction, this framework overcomes the inherent inefficiencies of conventional screening and provides a mechanistic rationale for the selection, characterization, and validation of novel bioactive resources such as *M. roseum*.

From a phytochemical perspective, *M. roseum* represents a distinct botanical resource compared with well-characterized antioxidant herbs such as Camellia, Ginkgo, and Cornus [58,59,60]. Unlike these glycoside-rich species, *M. roseum* exhibits an aglycone-dominant flavonoid profile, a feature that confers higher lipophilicity and improved cutaneous permeability [61]. Such physicochemical properties likely underlie the pronounced in vitro activity observed, despite the extract’s moderate intrinsic free-radical-scavenging capacity.

It was initially expected that iridoid glycosides or verbascoside-type phenylethanoids—bioactive constituents commonly reported in other members of the Orobanchaceae family and in genera such as Cornus, Gardenia, and Gentiana—would also contribute to the bioactivity profile of *M. roseum* [34,59,62,63]. However, these hydrophilic metabolites were largely absent from high-scoring protein–compound interaction pairs in the molecular docking and molecular-dynamics analyses, most likely due to poor compatibility with hydrophobic binding pockets. Their limited trans-epidermal diffusion further suggests restricted relevance for topical bioactivity, whereas aglycone derivatives such as loganetin or caffeic acid may play a role following oral administration or metabolic transformation.

Collectively, these results demonstrate the utility of combining high-resolution metabolomics with network-level computational analysis to accelerate natural-product discovery. The approach bridges empirical phytochemistry and molecular pharmacology, enabling quantitative mapping of multitarget effects. By elucidating how *M. roseum*-derived flavone aglycones modulate AKT1-, EGFR-, and COX-2-centered pathways, our work provides a concrete molecular framework that connects chemical identity to cutaneous biological function.

From a practical standpoint, the characterization of *M. roseum* as an aglycone-enriched botanical source suggests potential for formulation into dermocosmetic products. The dominance of small, lipophilic flavones indicates favorable skin permeability and stability, supporting their incorporation into UV-protective formulations without chemical enhancers. The reproducibility of this data-driven workflow further underscores its potential for systematic screening of regional flora and scalable industrial applications. This integrative strategy thus provides a template for translating computational prediction into practical, product-oriented innovation.

While the present study provides mechanistic insight into the anti-aging potential of *M. roseum*, several limitations warrant further investigation. Notably, all in vitro validations were conducted using the crude extract, not the individual metabolites prioritized by in silico analysis. While the bioactivities of individual constituents like apigenin and luteolin are well-documented [41,42], the specific contribution of each compound within this unique phytochemical matrix—and the potential for synergistic effects—remains to be experimentally quantified. Furthermore, experimental validation was confined to in vitro cell line models. Although this approach is suitable for the early-stage screening emphasized by our computational pipeline’s “from-selection-to-validation” framework, these models do not fully capture the complex interplay of immune, vascular, and barrier functions involved in cutaneous aging. Comprehensive in vivo studies employing UV-induced photoaging or oxidative-stress models are needed to confirm the physiological relevance, safety, and dermal penetration of the identified compounds.

From a biotechnological perspective, the hemiparasitic nature of *M. roseum* presents an additional challenge for large-scale utilization. Although the species can survive independently under controlled cultivation, it exhibits limited growth and diminished metabolite yield in the absence of a compatible host root system. This ecological dependency complicates sustainable biomass production for industrial applications. To enable sustainable production, future efforts should explore callus or hairy-root cultures, host-mimicking co-culture systems, and elicitor treatments such as methyl jasmonate or salicylic acid to enhance secondary metabolism. Parallel efforts to isolate key biosynthetic genes may also enable heterologous expression or metabolic engineering in tractable hosts. Addressing these challenges will be essential for translating the promising pharmacological profile of *M. roseum* into practical, scalable dermocosmetic applications.

## 4. Materials and Methods

### 4.1. Preparation of Melampyrum roseum Maxim. Extract

The plant extract (KPM011-018, code no: PMKR0044) used in this research was obtained from the Natural Product Central Bank at the Korea Research Institute of Bioscience and Biotechnology (Cheongju, Republic of Korea). A voucher specimen (KRIB 0001671) is kept in the herbarium of the Korea Research Institute of Bioscience and Biotechnology. The plant was collected from Taebaek-si, Gangwon-do, Republic of Korea in 2001. The plant (35 g) dried in the shade and powdered was added to 1 L of methyl alcohol 99.9% (HPLC grade) and extracted through 30 cycles (40 KHz, 1500 W, 15 min ultrasonication-120 min standing per cycle) at room temperature using an ultrasonic extractor (SDN-900H, SD-ULTRASONIC CO., LTD, Seoul, Republic of Korea). After filtering (Qualitative Filter No.100, HYUNDAI MICRO CO., LTD, Seoul, Republic of Korea) and drying under reduced pressure, *M. roseum* extract (3.42 g) was obtained.

### 4.2. UHPLC–MS/MS Analysis & Metabolite Identification

#### 4.2.1. UHPLC–MS/MS Analysis of *M. roseum* Extract

To perform qualitative analysis of the major compounds in the *M. roseum* extract, the sample was dissolved in methanol at a concentration of 1 mg/mL and filtered through a 0.2 μm PTFE syringe filter. UHPLC-MS/MS analysis was subsequently performed using a system combining an Ultra High Performance Liquid Chromatography (Vanquish Horizon UHPLC, Thermo Fisher Scientific, Waltham, MA, USA) with an Orbitrap ID-X Tribrid mass spectrometer (Thermo Fisher Scientific, Waltham, MA, USA). A sample injection volume of 1 μL was separated using a Hypersil GOLD™ Vanquish column (150 × 2.1 mm, 1.9 μm; Thermo Fisher Scientific, Waltham, MA, USA) maintained at a column oven temperature of 40 °C. The mobile phase consisted of Solvent A (Water containing 0.1% formic acid) and Solvent B (Acetonitrile containing 0.1% formic acid). Elution was performed at a flow rate of 0.3 mL/min using the following linear gradient: 0–3 min (5.0% B), 3–7 min (5.0–11.5% B), 7–8 min (11.5–12.0% B), 8–15 min (12.0–14.0% B), 15–25 min (14.0–25.0% B), 25–27 min (25.0–50.0% B), 27–29 min (50.0% B), 29–34 min (50.0–60.0% B), 34–36 min (60.0–100.0% B), 36–50 min (100.0% B), 50–55 min (100.0–5.0% B), and 55–60 min (5.0% B). MS analysis utilized a Heated Electrospray Ionization (H-ESI) source(Thermo Fisher Scientific, Waltham, MA, USA). The spray voltage was set to 3500 V in positive mode and 2500 V in negative mode. The flow rates for the Sheath gas, Aux gas, and Sweep gas were maintained at 50, 10, and 1 (arbitrary units), respectively. The ion transfer tube temperature was 320 °C, and the vaporizer temperature was set to 300 °C. Full MS scans were performed in the Orbitrap analyzer (Thermo Fisher Scientific, Waltham, MA, USA)at a high resolution of 120,000, covering the mass range of *m*/*z* 100–1500, with the RF lens set to 35%. MS/MS scans were acquired in data-dependent acquisition (DDA) mode at a resolution of 15,000. Fragmentation was induced using assisted Higher-energy Collisional Dissociation (HCD) with collision energies set to 30%, 40%, and 50%.

#### 4.2.2. Metabolite Identification

The raw data files were imported into MS-DIAL (v5.5.241113), an open-source software platform (https://systemsomicslab.github.io/compms/msdial/main.html) (accessed on 7 January 2025), for peak picking, deconvolution, deisotoping, alignment, and formula prediction. Metabolites were characterized based on their exact monoisotopic mass, retention time (tₘ), and MS/MS fragmentation patterns. Metabolite identification was performed by comparison with online chemical DBs (e.g., PubChem) and validated against reference literature, ensuring an acceptable confidence level (Level 2a: probable structure based on MS, MS/MS, and library/bibliography search) [64].

### 4.3. Reference-Based Identification of Potential Targets for Skin Aging

To define a reference set of molecular targets associated with skin aging, the term “skin aging” was used as a search query in the GeneCards and Open Targets Platform databases. Each database was queried independently to retrieve gene entries annotated with disease relevance. The resulting target lists were subsequently filtered according to the gene–disease association score, retaining only those genes exhibiting statistically significant or high-confidence associations with skin aging. Duplicate entries were merged based on official gene symbols to generate a non-redundant dataset for downstream network pharmacology analysis.

### 4.4. Metabolite Screening & Compound-Protein Interaction (CPI) Partner Prediction

Secondary metabolites identified by UHPLC–MS/MS analysis were subjected to computational screening to predict their putative molecular targets. To eliminate background interference from ubiquitous small molecules, primary metabolites such as sugars, amino acids, fatty acids, and nucleotides were excluded, and only structurally defined secondary metabolites were retained for further analysis. The canonical SMILES representation of each compound was retrieved from PubChem to ensure interoperability across target prediction platforms.

Information on experimentally validated compound–protein associations was extracted from the PubChem bioassay database, which integrates curated interaction data from individual DBs including Comparative Toxicogenomics Database (CTD), Drug–Gene Interaction Database (DGIdb), and BindingDB, as well as primary literature-derived bioassay results (PubChem Bioassay). To complement these curated datasets, potential targets were also predicted de novo using SwissTargetPrediction (version 2019) and STITCH v5.0, restricting species selection to Homo sapiens. For SwissTargetPrediction, only interactions with a probability score ≥ 0.1 were retained, while for STITCH, interactions with a combined confidence score ≥ 0.7 were considered high-confidence. Predicted and experimentally supported targets were merged, de-duplicated by UniProt accession number, and subsequently cross-referenced with the predefined skin aging–related gene set. The intersecting targets were defined as the bioactive target set of *M. roseum* for downstream network pharmacology and molecular docking analyses.

### 4.5. Network Pharmacology

#### 4.5.1. Protein-Protein Interaction Network Construction

To elucidate the pharmacological mechanisms underlying the anti–skin-aging effects of *M. roseum*, a compound–target–pathway interaction network was constructed. The validated and predicted compound–target pairs were imported into Cytoscape v3.10.4 to visualize the topological relationships between metabolites and their corresponding protein targets. The network was analyzed using the NetworkAnalyzer plugin to compute key topological parameters, including degree centrality, betweenness centrality, and closeness centrality, thereby identifying hub nodes with high regulatory significance.

The corresponding protein–protein interaction (PPI) network was generated using the STRING database v12.0, restricting the organism to Homo sapiens. Interactions with a confidence score ≥ 0.7 (high confidence) were retained, while disconnected nodes (‘singletons’) were removed. The resulting network was imported into Cytoscape for visualization and further analysis.

#### 4.5.2. Pathway Enrichment Analysis

To characterize the biological functions of the core targets, Gene Ontology (GO) enrichment—including biological process (BP), cellular component (CC), and molecular function (MF) categories—, and Kyoto Encyclopedia of Genes and Genomes (KEGG) pathway enrichment analyses were performed using the ShinyGO 0.85 platform. Enrichment significance was determined by a false discovery rate (FDR) < 0.05 and gene count ≥ 3. Redundant or semantically overlapping GO terms were clustered using ShinyGO’s hierarchical grouping plot option. The top 15 GO terms and KEGG pathways were selected according to adjusted *p*-values and visualized consequently.

### 4.6. Molecular Dynamics

#### 4.6.1. Protein Structure Preparation

To perform molecular docking and dynamics analyses, protein structures corresponding to the top-ranked target genes—including both high-scoring protein–compound interaction candidates and hub genes identified from the *M. roseum* target network—were retrieved from the Protein Data Bank (PDB) (Supplementary Table S12). Preference was given to crystal structures complexed with small-molecule inhibitors or co-crystallized ligands whenever available (Table S1). Crystal structures were collected for sixteen target proteins, comprising STAT3, AKT1 (ATP-binding and allosteric sites), MMP9, TNF-α, PTGS2, EGFR (ATP-binding and allosteric sites), RELA, MAPK3, IL1B, MAPK14, AMPK, CASP3, GSK3B, PPARG, XDH, and NOS2. All utilized PDB structures are listed in Supplementary Table S12.

All protein structures were prepared using the Protein Preparation Wizard in Maestro (Schrödinger, LLC, New York, NY, USA, version 2025-1). Missing loops or side chains were modeled using Prime(Schrödinger, LLC, New York, NY, USA, version 2025-1), and hydrogen atoms were added considering physiological pH (7.4). Protonation states of titratable residues were adjusted with PROPKA (pH 7.4), followed by optimization of hydrogen bonding networks.

Restrained energy minimization was carried out using the OPLS4 force field with a heavy-atom convergence threshold of 0.3 Å RMSD. Crystallographic water molecules located beyond 5 Å from the native ligand binding sites were removed, while those potentially involved in ligand stabilization were retained for subsequent docking calculations. All structural preparation settings were kept consistent across the 18 target complexes to ensure uniformity in downstream docking and simulation analyses.

#### 4.6.2. Molecular Docking

Molecular docking was conducted to generate binding poses using Glide (Maestro, Schrödinger LLC, New York, NY, USA, version 2025-1). The receptor grid generation module in Maestro was used to define the protein–ligand docking sites. Ligands were docked in standard precision (SP) mode against all target proteins.

For seventeen of the target structures, docking sites were defined based on the co-crystallized ligands present in the corresponding PDB complexes. In the case of RELA, which lacks a canonical small-molecule binding site and instead binds to DNA, two approaches were employed to determine a feasible binding pocket:

- Selection of the region corresponding to the DNA-binding interface, and
- Identification of potential druggable pockets using the SiteMap module in Maestro.

All docking settings were applied uniformly across the targets to ensure consistency in pose generation and scoring.

#### 4.6.3. MD Simulation

Molecular dynamics (MD) simulations were carried out using Desmond in Maestro (Schrödinger, LLC, New York, NY, USA, version 2025-1). Eight protein–ligand complexes were selected for simulation, consisting of Chrysoeriol, Luteolin, and Apigenin bound to PTGS2, EGFR allosteric site, AKT1 allosteric site, and XDH, based on their favorable docking scores and key binding interactions.

Each complex was solvated in an orthorhombic TIP3P water box with a 10 Å buffer from the solute. Counterions (Na^+^ and Cl^−^) were added to neutralize the system and to maintain physiological salinity (0.15 M). The OPLS4 force field was applied to describe protein–ligand interactions. All simulations were performed under NPT ensemble conditions at 300 K and 1.01325 bar using the Nose–Hoover thermostat and Martyna–Tobias–Klein barostat. System equilibration was carried out following standard Desmond relaxation protocols [65].

For each of the eight complexes, three independent 100 ns simulation replicas were performed using distinct random seeds (2007, 4222, and 9177) to ensure reproducibility and statistically reliable sampling. Trajectory analyses were performed using the Simulation Interaction Diagram tool in Maestro. Protein backbone atoms were aligned to the initial frame prior to calculating Protein Cα RMSD to evaluate global protein conformational stability. Ligand RMSD was assessed to monitor ligand positional fluctuations within the binding pocket during the simulation.

*RMSD* values were calculated based on Equation (1):

(1) $${RMSD}_{X}=\sqrt{\frac{1}{N}\sum_{i=1}^{N}{\left({r^{\prime }}_{i}\left(t_{x}\right)\right)-r_{i}(t_{ref}))}^{2}}\;$$

where *N* represents the number of atoms, *t_ref_* is the reference time, *r*^′^ denotes the coordinates of atoms at time *t_x_* after least-squares superposition on the reference frame, and *t_x_* is the simulation time of the corresponding analyzed frame.

Protein Cα RMSD values within 1–3 Å were considered indicative of stable protein structures, while ligand RMSD was used to assess binding retention throughout the simulation.

### 4.7. Experimental Validation

#### 4.7.1. MTT Viability Assay

RAW264.7 cells (3 × 10^5^ cells/mL) and Human Dermal Fibroblast (HDF) cells (1 × 10^5^ cells/mL) were individually seeded into 96-well plates. The cells were stabilized by incubation for 24 h at 37 °C in a 5% CO_2_ incubator. The culture medium was then removed, and the cells were re-incubated for another 24 h in a CO_2_ incubator after adding 180 μL of FBS-free DMEM containing 1% P/S and 20 μL of the sample diluted to the appropriate concentration. Following the incubation period, the attached cells were treated for 30 min with a solution prepared by mixing 20 μL of 5 mg/mL MTT (3-(4,5-dimethylthiazol-2-yl)-2,5-diphenyltetrazolium bromide) solution with 980 μL of serum-free medium. The MTT solution was then removed, and 100 μL of DMSO was added. The plate was shaken for 1 min on a plate shaker, and the absorbance was measured at 550 nm. The results were calculated as cell viability (%) relative to the untreated control group.

#### 4.7.2. Radical Scavenging Assay

The antioxidant capacity of *M. roseum* extract was assessed using ABTS and DPPH radical scavenging assays. For the ABTS assay, the ABTS•^+^ radical cation was generated by mixing 14 mM ABTS solution (prepared in 1× PBS) with 4.9 mM potassium persulfate at a 1:1 (*v*/*v*) ratio and allowing the reaction to proceed in the dark at room temperature for 12 h. The resulting ABTS•^+^ solution was diluted with PBS to achieve an absorbance of 0.70 ± 0.10 at 734 nm. Subsequently, 10 μL of the extract, diluted to the designated concentrations in PBS, was mixed with 190 μL of the ABTS•^+^ working solution in a 96-well plate. After 6 min incubation at room temperature, the absorbance was measured at 734 nm using a SpectraMax iD3 microplate reader (Molecular Devices, San Jose, CA, USA). Ascorbic acid (AA) served as a positive control, and PBS alone was used as the blank. The radical scavenging activity (%) was calculated relative to the control group. For the DPPH assay, a 0.3 mM DPPH solution was prepared in 80% methanol and equilibrated for 30 min in the dark prior to use. Each well received 10 μL of appropriately diluted sample and 190 μL of DPPH working solution. The mixture was incubated for 30 min in the dark at room temperature, and the absorbance was recorded at 517 nm using the same microplate reader. Ascorbic acid and 80% methanol were used as the positive and negative controls, respectively. The DPPH radical scavenging activity (%) was determined by comparing the absorbance of treated wells against that of the control.

#### 4.7.3. Anti-Inflammatory Activity Assay

The anti-inflammatory activity of *M. roseum* extract was evaluated in murine macrophage RAW 264.7 cells. Cells were seeded at a density of 3 × 10^5^ cells/mL in 12-well plates and incubated at 37 °C in a humidified atmosphere containing 5% CO_2_ for 24 h. After stabilization, the culture medium was replaced with FBS-free DMEM containing 1% penicillin–streptomycin (P/S) and the extract diluted to the indicated concentrations. Cells were pre-treated with the extract for 30 min, after which inflammation was induced by the addition of 10 μg/mL lipopolysaccharide (LPS). Following 24 h incubation, either cultured cell or culture supernatant were collected for subsequent analyses.

Levels of pro-inflammatory cytokines (IL-6 and TNF-α) were quantified using enzyme-linked immunosorbent assays (ELISA) according to the manufacturer’s instructions, and absorbance was measured at 450 nm. The inhibitory effect of the extract was expressed relative to the LPS-treated control group. Nitric oxide (NO) production in the same supernatants was determined using the Griess reagent, and absorbance was read at 540 nm to calculate the percentage inhibition of NO formation. The expression level of cyclooxygenase-2 (COX-2) was analyzed by quantitative real-time PCR (qRT-PCR). Total RNA was extracted using Easy-Blue™ reagent, and cDNA was synthesized from 1 μg of RNA with the iScript™ cDNA Synthesis Kit. qRT-PCR was performed using TB Green Premix Ex Taq with gene-specific primers for COX-2 and the internal control GAPDH. Relative expression levels were calculated by the 2^−^ΔΔCt method, and the results were compared between sample-treated and untreated control cells to assess the transcriptional suppression of COX-2.

#### 4.7.4. Anti-Photoaging Effect Assessment (MMP-1 Assay)

HDF cells (1 × 10^5^ cells/mL) were seeded into a 12-well plate and stabilized by incubation at 37 °C in a 5% CO_2_ incubator for 24 h. Subsequently, the culture medium was removed, and a wrinkle-inducing environment was established by adding 400 μL of HBSS and irradiating with UVB (20 mJ cm^−2^). The HBSS was then removed, and the cells were re-incubated for 24 h in the CO_2_ incubator after adding 900 μL of FBS-free DMEM medium containing 1% P/S and 100 μL of the sample diluted to appropriate concentrations. Following the incubation period, the culture supernatant was collected, and the production level of MMP-1 was quantified by performing ELISA. The absorbance was measured at 450 nm and compared with the untreated control group.

## 5. Conclusions

In summary, this study establishes a comprehensive framework for data-driven natural-product discovery by integrating metabolomics, computational modeling, and experimental validation. Using this multi-layered analytical pipeline, we systematically characterized *Melampyrum roseum*, a previously uncharacterized East Asian herb, revealed a diverse metabolome enriched in flavonoids, iridoids, and phenylethanoid glycosides. Network pharmacology and molecular-dynamics simulations identified critical interactions between these metabolites and key regulators of oxidative and inflammatory signaling—such as AKT1, EGFR, PTGS2, and MMP-1—implicating the PI3K–Akt, MAPK, and ECM-degradation pathways. Consistent with in silico predictions, *M. roseum* extracts exhibited potent antioxidant and anti-inflammatory effects in UVB-exposed human dermal fibroblasts without detectable cytotoxicity.

Collectively, our findings highlight *M. roseum* as a novel botanical reservoir of multifunctional metabolites capable of modulating the molecular cascades underlying skin aging and underscore the potential of the integrative informatics-to-bioassay pipeline for accelerating big data-guided rational natural product research.

## Figures and Tables

**Table 1:** 13 major secondary metabolites identified in *M. roseum* extract.

No.	Molecule Name	RT	Monoisotopic Mass	Molecular Formula	Intensity (POS)	Intensity (N × 10 G)
3	Aucubin	2.47	346.1264	C_15_H_22_O_9_	ND	1.33 × 10^6^
4	Geniposidic acid	3.05	374.1213	C_16_H_22_O_10_	ND	1.62 × 10^6^
5	Loganic acid	6.27	393.1634	C_16_H_24_O_10_	2.56 × 10^6^	1.39 × 10^7^
6	Mussaenosidic acid	6.85	393.1634	C_16_H_24_O_10_	3.73 × 10^5^	3.20 × 10^6^
7	Secologanin	7.59	388.1369	C_17_H_24_O_10_	7.26 × 10^5^	5.92 × 10^6^
10	Loganin	10.3	390.1514	C_17_H_26_O_10_	2.99 × 10^6^	1.48 × 10^7^
11	Mussaenoside	11.38	390.1514	C_17_H_26_O_10_	5.94 × 10^6^	5.53 × 10^7^
14	Hyperoside	18.1	464.0953	C_21_H_20_O_12_	1.06 × 10^6^	1.48 × 10^6^
15	(iso)verbascoside	20.31	624.2054	C_29_H_36_O_15_	1.80 × 10^6^	1.01 × 10^7^
16	Apigenin glucuronide	22.53	446.0849	C_21_H_18_O_11_	6.26 × 10^5^	1.80 × 10^6^
17	Luteolin	27.14	286.0472	C_15_H_10_O_6_	3.36 × 10^6^	6.94 × 10^6^
18	Apigenin	28.37	270.0522	C_15_H_10_O_5_	2.21 × 10^7^	2.72 × 10^7^
19	Chrysoeriol	28.55	300.0627	C_16_H_12_O_6_	2.46 × 10^6^	3.58 × 10^6^

ND: Not detected.

## Data Availability

Publicly available datasets were analyzed in this study. Disease–gene/protein relationship data were obtained from GeneCards (https://www.genecards.org/ (accessed on 5 December 2025)) and the Open Targets Platform (https://platform.opentargets.org/ (accessed on 5 December 2025)). Protein–protein interaction (PPI) data were retrieved from the STRING database (https://string-db.org/ (accessed on 5 December 2025)). Experimentally validated protein–chemical interaction (PCI) data were sourced from PubChem (https://pubchem.ncbi.nlm.nih.gov/ (accessed on 5 December 2025)), while bibliometric statistics for specific compounds were derived from PubMed (https://pubmed.ncbi.nlm.nih.gov/ (accessed on 5 December 2025)) and Springer Nature (accessed via PubChem). Prediction of PCIs was performed using SwissTargetPrediction (https://www.swisstargetprediction.ch/ (accessed on 5 December 2025)) and STITCH (http://stitch.embl.de/ (accessed on 5 December 2025)). Gene enrichment analysis was conducted using ShinyGO 0.85 (https://bioinformatics.sdstate.edu/go/ (accessed on 5 December 2025)). All data subsets retrieved and analyzed during the current study are available in the Supplementary Materials (Tables S2–S11).

## Supplementary Materials

The following supporting information can be downloaded at: https://www.mdpi.com/article/10.3390/ijms262411853/s1.

## Abbreviations

The following abbreviations are used in this manuscript:

CPI	Compound-protein interaction
ECM	Extracellular matrix
GO	Gene ontology
HDF	Human dermal fibroblast
MD	Molecular dynamics
NP	Natural product
PPI	Protein-protein interaction
RMSD	Root mean square distance
ROS	Reactive oxygen species
UHPLC	Ultra high performance liquid chromatography
UV	Ultraviolet
