# Supplementary material for: A Multi-Layered Analytical Pipeline Combining Informatics, UHPLC–MS/MS, Network Pharmacology, and Bioassays for Elucidating the Skin Anti-Aging Activity of Melampyrum roseum
Source: Int J Mol Sci. 2025 Dec 8;26(24):11853. doi: 10.3390/ijms262411853 (PMC12733246; doi:10.3390/ijms262411853)
Supplement: Supplementary file 1 [file ijms-26-11853-s001.zip › IJMS_CHO_article_supplementary_material.pdf]

Supplementary material

A multi-layered analytical pipeline combining informatics, UHPLC–MS/MS, network pharmacology, and bioassays for elucidating the skin anti-aging activity of *Melampyrum roseum*

Min Hyung Cho[1*], JangHo Ha[1,2], Haiyan Jin[1], SoHee An[1], SungJune Chu[1]

1 Bioinformatics and Molecular Design Research Center (BMDRC). Songdogwahak-ro 85, Yeonsu-gu, In-cheon, 21983, Republic of Korea

2 Department of Integrative Biotechnology, Yonsei University, Songdogwahak-ro 85, Yeonsu-gu, Incheon, 21983, Republic of Korea

* Correspondence: Min Hyung Cho (mhcho@bmdrc.org)

Contents

Supplementary figures

Supplementary figure S1. Comparison of LC profiles between independently collected M. roseum samples.

Supplementary figure S2. Comparison of observed MS/MS profiles with reference compound MS/MS patterns.

Supplementary figure S3. Additional pathway enrichment analysis results involving 122 intersecting genes. (A) GO biological processes (BP)-based analysis. (B) GO cellular components (CC) -based analysis.

Supplementary figure S4. Additional pathway enrichment analysis results involving 172 *M. roseum* targets. (A) GO biological processes (BP)-based analysis. (B) GO cellular components (CC) -based analysis.

Supplementary figure S5. Molecular dynamics simulation results of three compounds bound to target proteins (replicate 2)

Supplementary figure S6. Molecular dynamics simulation results of three compounds bound to target proteins (replicate 3)

Supplementary figure S7. Protein–ligand contact profiles for three compounds bound to target proteins.

Supplementary tables (in separate spreadsheet)

Supplementary table S1. Full list of identified metabolites from *M. roseum* extract

Supplementary table S2. Full list of skin aging-related genes identified from Genecards & Open Targets databases

Supplementary table S3. Detailed information of PPI network consists of 122 intersecting genes

Supplementary table S4. Full pathway enrichment analysis results involving 122 intersecting genes

Supplementary table S5. Full list of M. roseum metabolite-protein association annotations collected from PubChem pre-compiled dataset

Supplementary table S6. Full list of M. roseum metabolite-protein association annotations collected from PubChem bioassay dataset

Supplementary table S7. Full list of M. roseum metabolite-protein association prediction results produced with SwissTargetPrediction

Supplementary table S8. Full list of M. roseum metabolite-protein association prediction results produced with STITCH

Supplementary table S9. Detailed information of PPI network consists of 172 *M. roseum*-associated skin aging targets

Supplementary table S10. Full pathway enrichment analysis results involving 172 *M. roseum* targets

Supplementary table S11. Summary of protein target structures and corresponding PDB IDs used for molecular docking analysis.

Supplementary table S12.

Supplementary table S13.

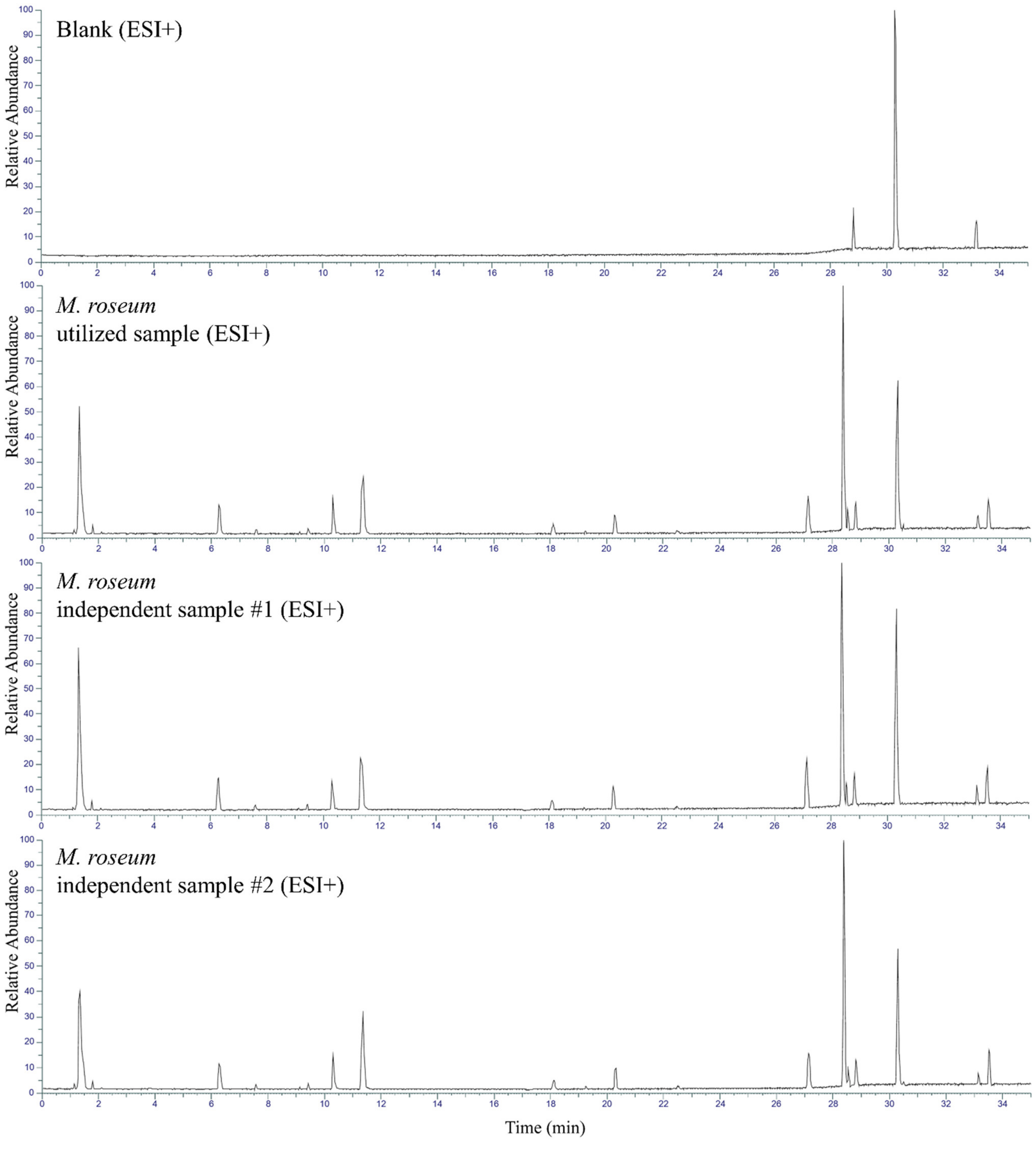

(continued in next page)

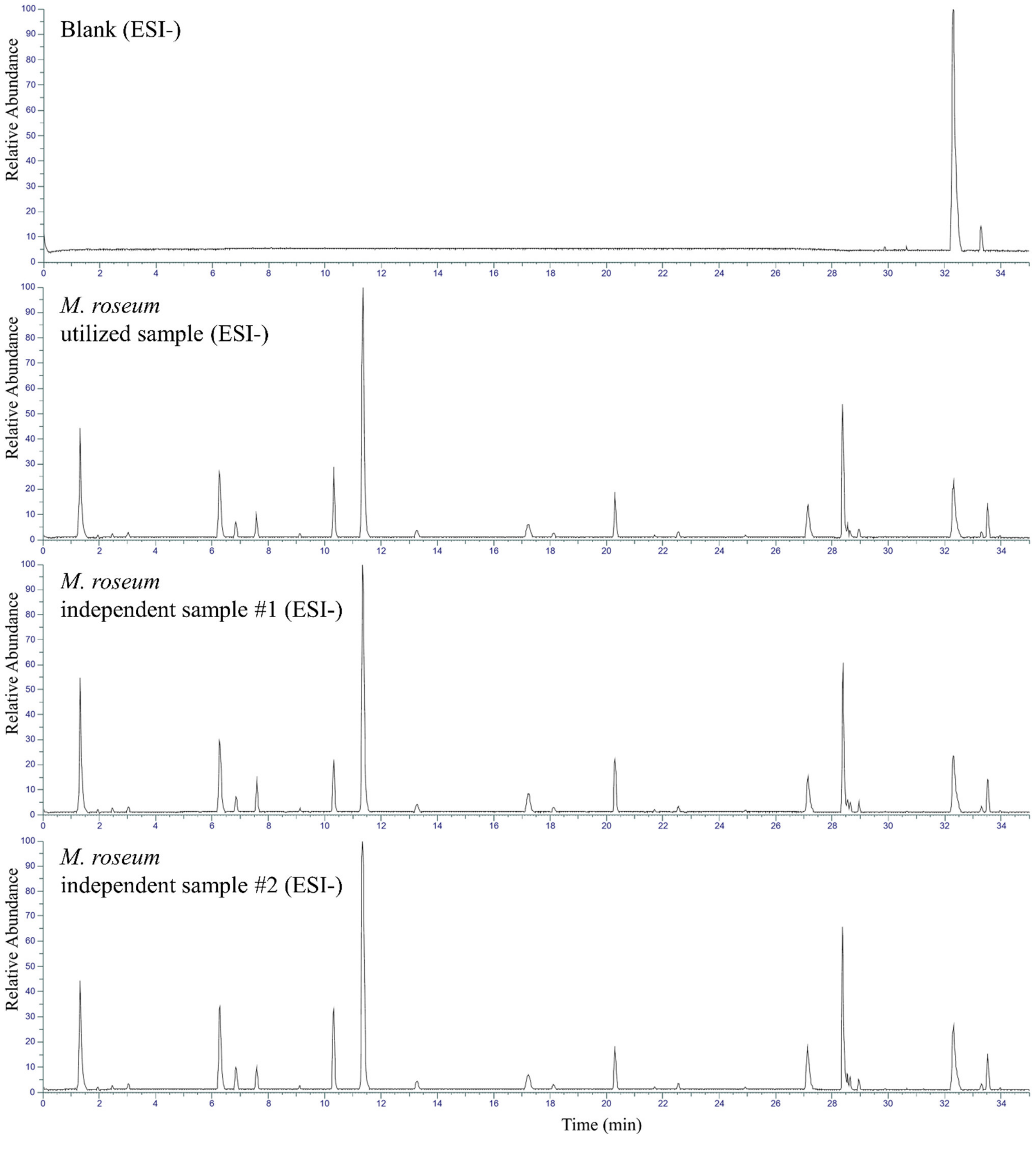

Supplementary figure S1. Comparison of LC profiles between independently collected M. roseum samples. (Upper panel): ESI+ (positive ionization mode). (Lower panel): ESI- (negative ionization mode).

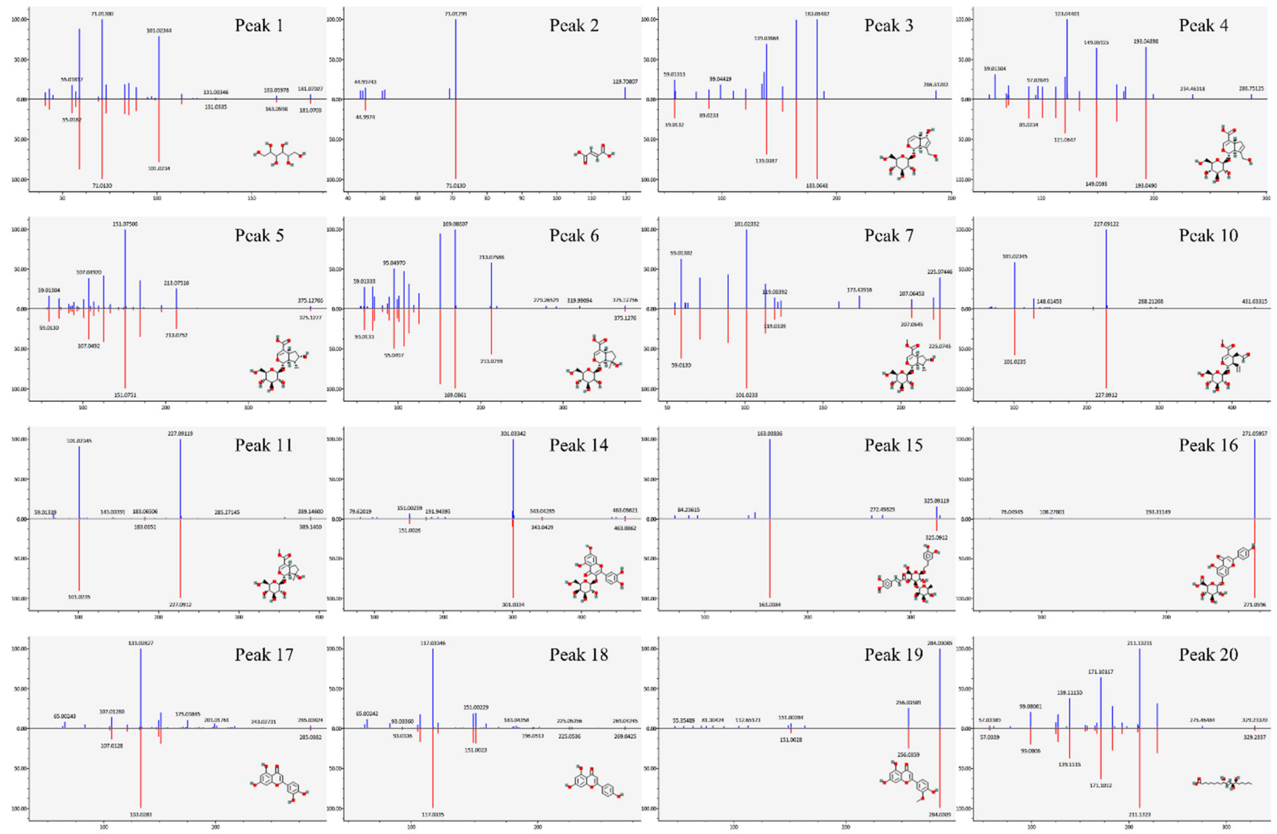

Supplementary figure S2. Comparison of observed MS/MS profiles with reference compound MS/MS patterns. Blue traces represent the experimentally observed MS/MS spectra for each peak (peak numbers indicated in the upper right), whereas red traces represent the reported MS/MS spectra for the corresponding reference compounds (chemical structures shown in the lower right).

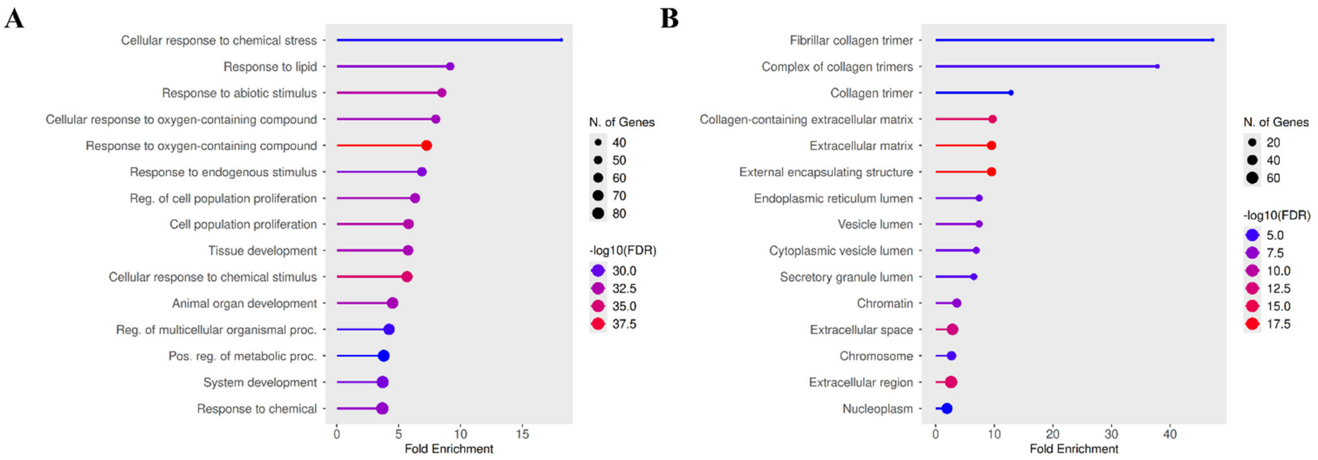

Supplementary figure S3. Additional pathway enrichment analysis results involving 122 intersecting genes. **(A)** GO biological processes (BP)-based analysis. **(B)** GO cellular components (CC) -based analysis.

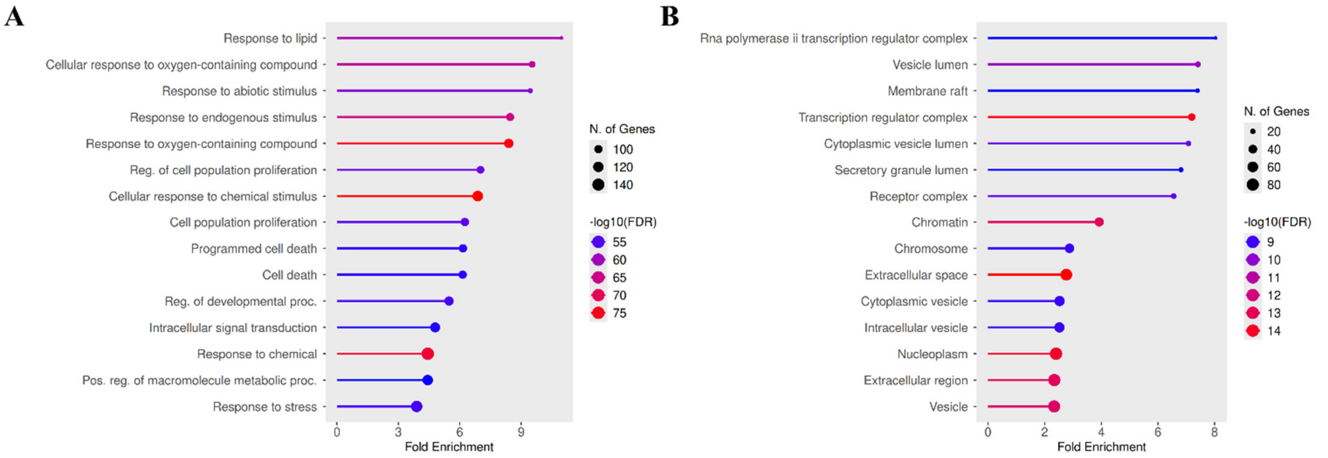

Supplementary figure S4. Additional pathway enrichment analysis results involving 172 *M. roseum* targets. **(A)** GO biological processes (BP)-based analysis. **(B)** GO cellular components (CC) -based analysis.

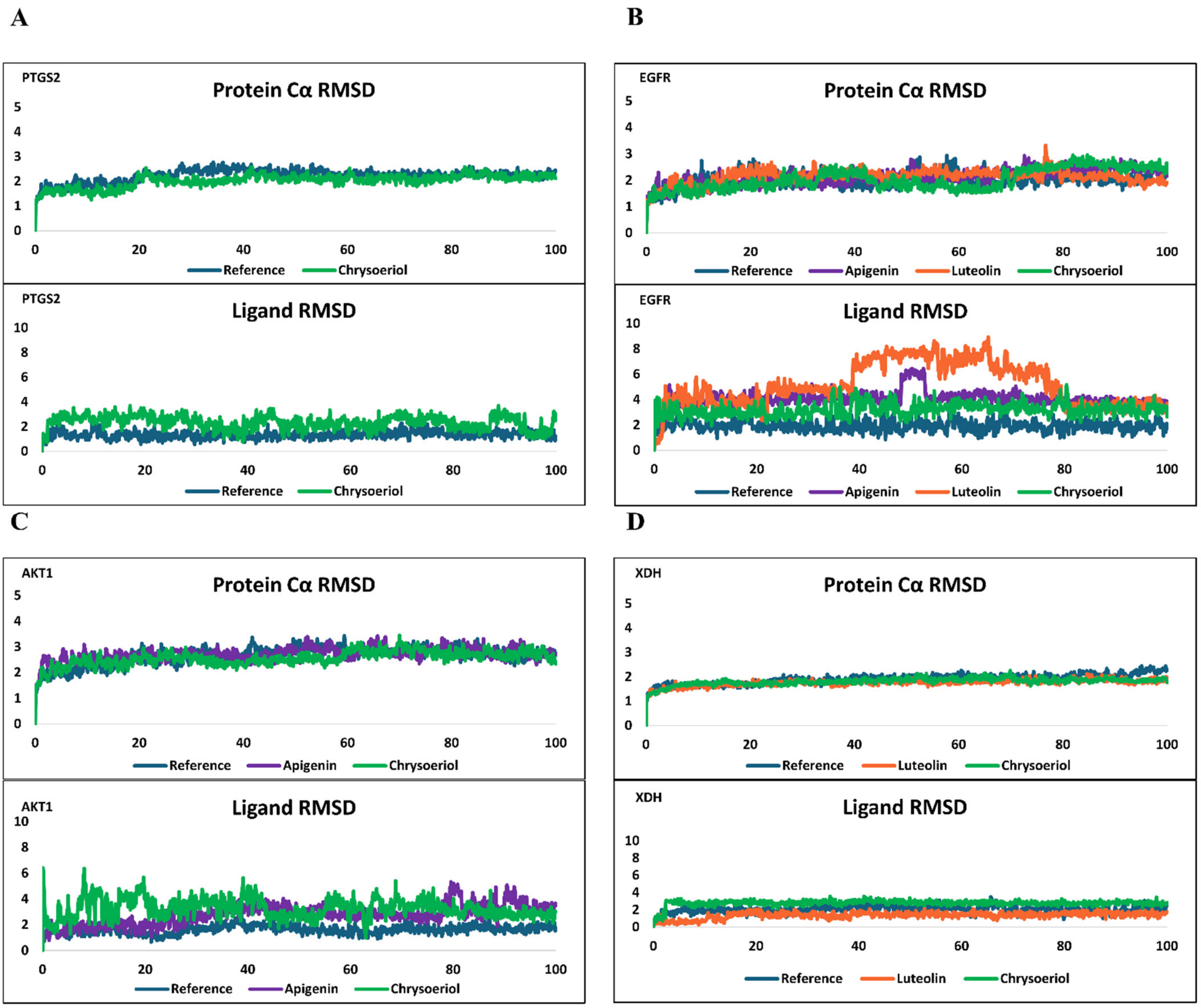

Supplementary figure S5. MD simulation results of select compound-protein pairs (replicate 2). Protein Cα and ligand RMSD trajectories over the 100 ns simulation are presented for the protein–ligand complexes. The reference ligand corresponds to the co-crystallized ligand from each target protein structure. Chrysoeriol, Luteolin, and Apigenin are represented in green, orange, and purple traces, respectively. **(A)** PTGS2. **(B)** EGFR. **(C)** AKT1. **(D)** XDH.

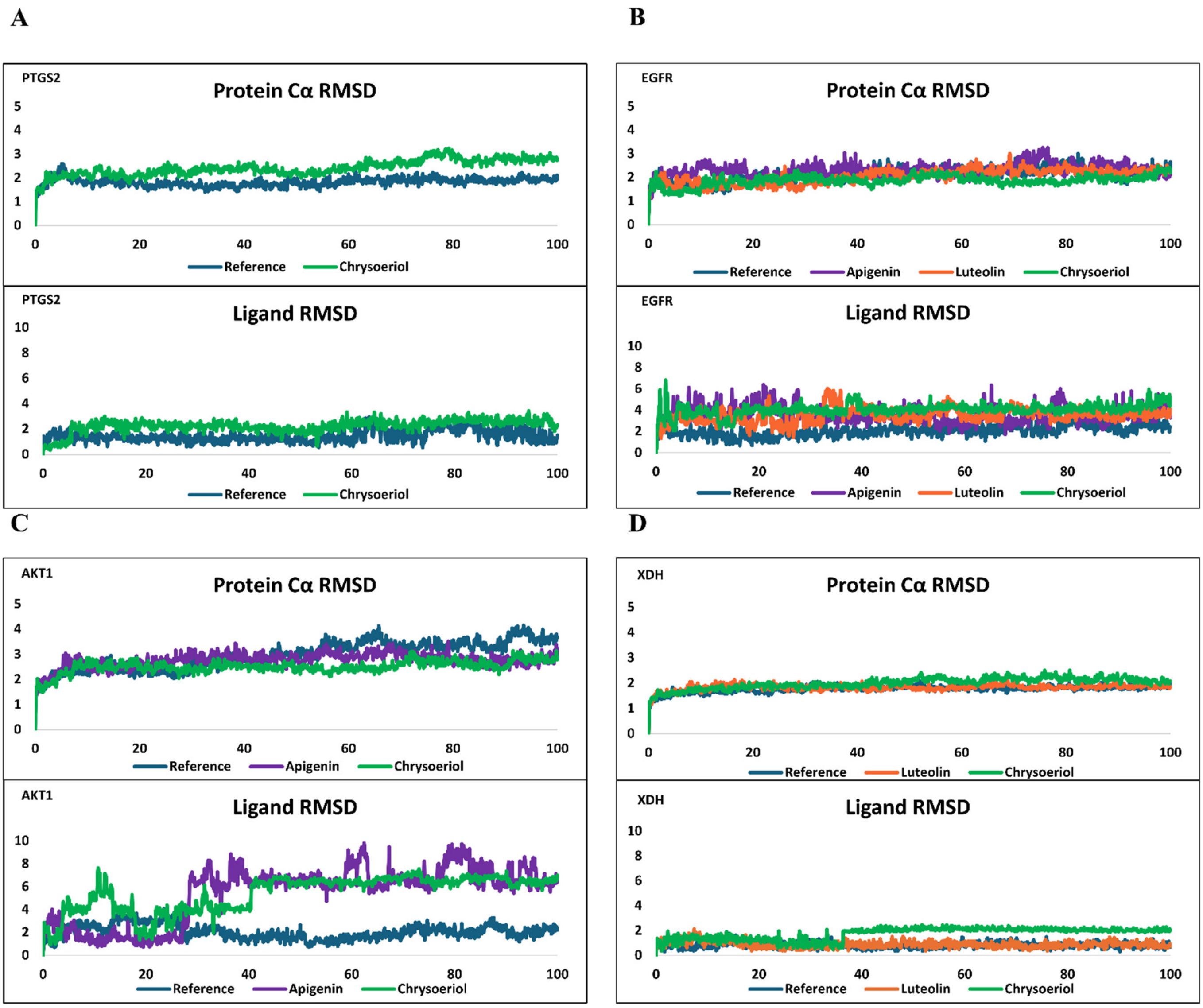

Supplementary figure S6. MD simulation results of select compound-protein pairs (replicate 3). Protein C*α* and ligand RMSD trajectories over the 100 ns simulation are presented for the protein–ligand complexes. The reference ligand corresponds to the co-crystallized ligand from each target protein structure. Chrysoeriol, Luteolin, and Apigenin are represented in green, orange, and purple traces, respectively. **(A)** PTGS2. **(B)** EGFR. **(C)** AKT1. **(D)** XDH.

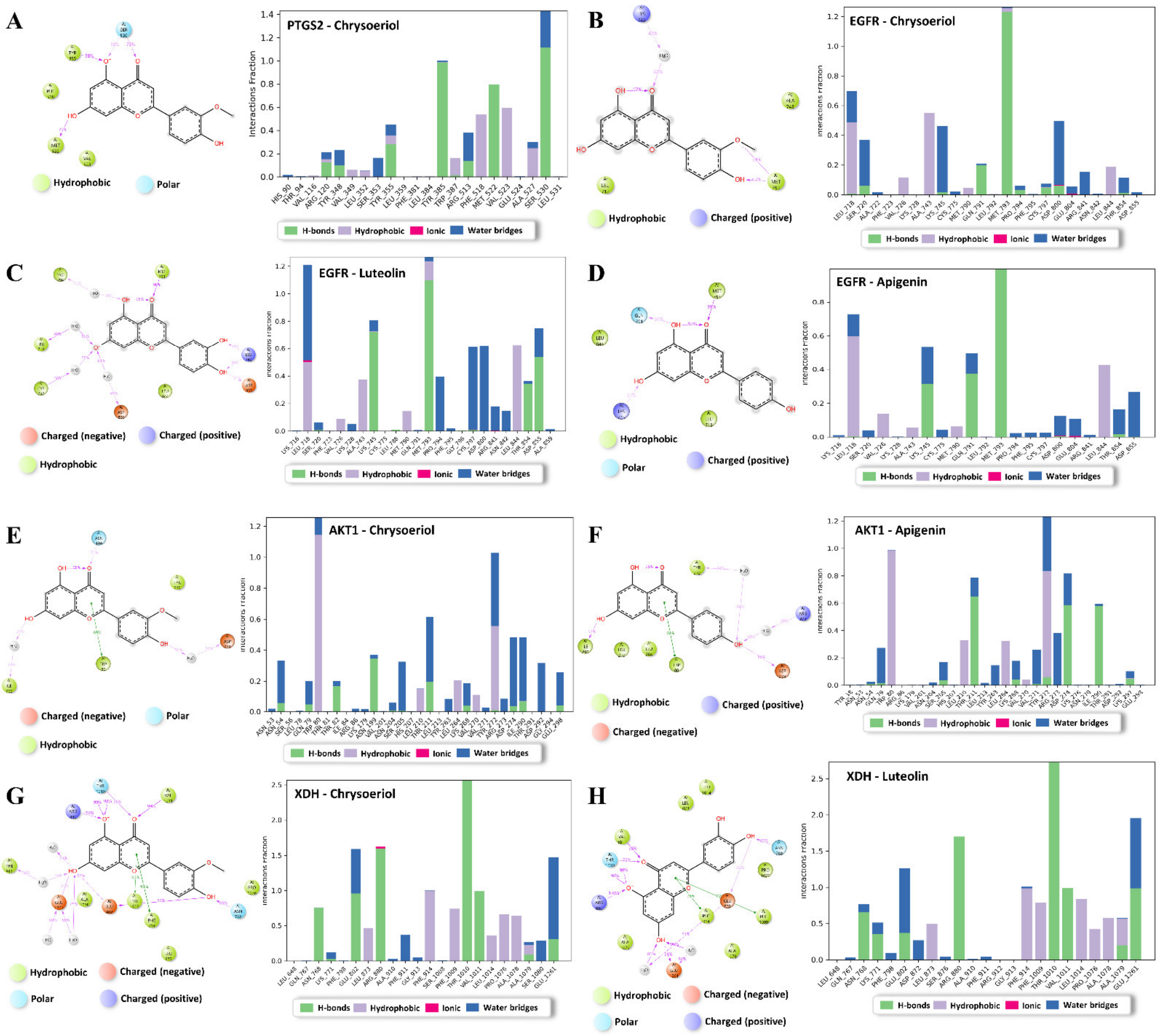

Supplementary figure S7. Protein–ligand contact profiles for three compounds bound to target proteins. The upper panel of each subfigure displays the 2D interaction diagram, summarizing key contacts formed between the ligand and the target protein. Only interactions that persist for more than 30% of the total simulation time are shown. The lower panel illustrates the corresponding protein–ligand interaction fraction histogram throughout the simulation.
